# Non-Bovine Milk as Functional Foods with Focus on Their Antioxidant and Anti-Inflammatory Bioactivities

**DOI:** 10.3390/antiox14070801

**Published:** 2025-06-27

**Authors:** Yan Li, Qingshan Ma, Mengmeng Li, Wenqiang Liu, Yihong Liu, Menghan Wang, Changfa Wang, Muhammad Zahoor Khan

**Affiliations:** College of Agriculture and Biology, Liaocheng University, Liaocheng 252000, China

**Keywords:** camel milk, donkey milk, antioxidant and anti-inflammatory activities, bioactive compounds, functional foods, therapeutic potential

## Abstract

The growing interest in functional foods has directed scientific attention toward alternative milk sources, particularly camel and donkey milk, which have been traditionally consumed for their purported health benefits across diverse cultures. These milk sources possess unique nutritional profiles and bioactive compositions that differ substantially from conventional bovine milk. This review examines the current scientific understanding of the anti-inflammatory and antioxidant bioactivities of camel and donkey milk, exploring their bioactive constituents and therapeutic potential. Camel and donkey milk demonstrate notable antioxidant and anti-inflammatory properties that may exceed those of conventional milk sources. Key bioactive compounds include lactoferrin, lysozyme, immunoglobulins, bioactive peptides, vitamins C and E, and polyunsaturated fatty acids. Mechanistic studies reveal that milk from donkeys and camels suppresses inflammatory pathways through NF-κB inhibition, cytokine modulation (reducing IL-6, IL-1β, and TNF-α while enhancing IL-10), and antioxidant pathway activation via Nrf2-ARE signaling. Donkey milk exhibits particularly high lysozyme content and demonstrates significant immunomodulatory effects, while camel milk shows remarkable therapeutic potential in diabetes management, nephroprotection, and hepatoprotection. Preclinical studies demonstrate efficacy in treating oxidative stress-related disorders, inflammatory conditions, metabolic dysfunction, and tissue injury models. Altogether, the published data show that camel and donkey milk represent promising functional foods with significant antioxidant and anti-inflammatory bioactivities mediated through multiple molecular pathways. Their unique bioactive profiles offer therapeutic potential for various health conditions, warranting further clinical investigation and development as nutraceutical interventions.

## 1. Introduction

Milk is a white liquid produced by female mammals to nourish their young until they develop the ability to consume solid food. It is a complex biological fluid containing a variety of nutrients and bioactive components essential for health, growth, and development [1]. Dairy products represent a rich reservoir of bioactive peptides, making them attractive options for those seeking natural alternatives to conventional medications. In recent years, the scientific community has witnessed a growing interest in alternative milk sources beyond conventional bovine milk, with camel and donkey milk emerging as a promising functional food with unique bioactive properties [2,3,4,5]. These types of milk have been traditionally consumed in various regions worldwide for their purported health benefits, ranging from improved immunity to enhanced healing of various ailments [6,7,8]. While their traditional use spans centuries across diverse cultures in Africa, the Middle East, and parts of Asia, only recently have systematic biochemical analyses and molecular studies begun to elucidate the scientific basis for their therapeutic potential [1,6,9,10,11].

The remarkable nutritional composition of camel and donkey milk differs substantially from that of more common dairy sources [12,13,14,15,16]. These differences extend beyond basic macronutrient profiles to include distinctive bioactive compounds with potential health-promoting effects [15,17,18]. Of particular interest are their antioxidant and anti-inflammatory properties, which have garnered significant attention due to their implications in combating oxidative stress and inflammation—two fundamental processes underlying numerous chronic diseases, including cardiovascular disorders, diabetes, neurodegenerative conditions, and various inflammatory pathologies [9]. Concurrently, their anti-inflammatory bioactivity appears to operate through multiple pathways, modulating cytokine production, inhibiting inflammatory enzymes, and regulating immune cell function [19,20]. These dual protective mechanisms may explain the broad spectrum of health benefits attributed to camel and donkey milk consumption in both traditional knowledge and emerging clinical observations [6,7,8,21]. Antioxidant compounds in donkey and camel milk, including vitamins C and E, selenium, zinc, lactoferrin, and various peptides, function by neutralizing reactive oxygen species and preventing oxidative damage to cellular components [4,22]. In addition, the antioxidant properties of lactoferrin are well documented in previous studies [23].

**Figure 1:**
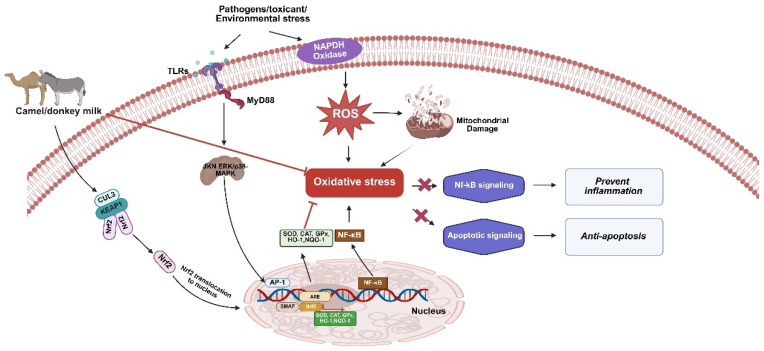
Antioxidant and anti-inflammatory potential of camel and donkey milk. This figure illustrates the comprehensive protective mechanism by which camel and donkey milk components counteract cellular damage caused by oxidative stress. Environmental stressors, pathogens, and toxicants enter the cell through toll-like receptor 4 (TLR4) and activate myeloid differentiation factor 88 (MyD88) pathway, leading to the generation of reactive oxygen species (ROS). ROS production results in mitochondrial damage and triggers oxidative stress, which normally would lead to cellular dysfunction and apoptosis. Camel and donkey milk components intervene in this cascade by modulating key signaling pathways—specifically inhibiting nuclear factor kappa-light-chain-enhancer of activated B cells (NF-κB) signaling to reduce inflammation and regulating apoptotic signaling to promote anti-apoptotic responses. The activation of nuclear factor erythroid-2 related factor 2 (Nrf2) signaling leads to enhanced antioxidant responses, which suppress oxidative stress and consequent damage.

The antioxidant functionality of camel and donkey milk appears to be mediated through the nuclear factor erythroid-2 related factor 2 (Nrf2) signaling pathway, which regulates antioxidant function throughout the body. Previous research has suggested that donkey and camel milk’s antioxidant properties may be attributed to stimulation of the Nrf2- antioxidant response element (Nrf2-ARE) pathway, as shown in Figure 1 [1,6,9,24,25,26,27]. The Nrf2 represents a crucial transcription factor that plays a significant role in anti-inflammatory and antioxidant stress responses. Under normal physiological conditions, Nrf2 predominantly resides within the cytoplasm, forming a complex with Kelch-like ECH-associated protein 1 (Keap1) [28,29,30,31]. The Keap1 functions as a negative regulator of Nrf2, mediating its ubiquitination and subsequent proteasomal degradation, thereby maintaining Nrf2’s physiological activity at lower levels. When cellular systems are exposed to inflammation or oxidative stress, Nrf2 undergoes phosphorylation and dissociates from Keap1, leading to its induction. Subsequently, activated Nrf2 translocates into the cell nucleus, dimerizes with small musculoaponeurotic fibrosarcoma proteins (sMafs), and binds to AREs within the promoter regions of relevant antioxidant genes. Upon Nrf2-induced ARE activation, downstream target genes are regulated, resulting in increased synthesis of glutathione peroxidase (GSH-Px) and superoxide dismutase (SOD), ultimately promoting enhanced antioxidant capacity throughout the organism [30,31,32]. This cascade of events enhances antioxidant defense mechanisms while concurrently modulating downstream inflammatory pathways. The upregulation of antioxidant gene expression attenuates excessive reactive oxygen species (ROS) generation, thereby preventing ROS-mediated activation of nuclear factor kappa B (NF-κB) signaling and mitochondrial dysfunction. This regulatory mechanism is critical, as both NF-κB pathway activation and mitochondrial damage serve as key drivers of pro-inflammatory cytokine production and subsequent inflammatory responses (Figure 1) [32,33,34,35].

Given the fundamental importance of these antioxidant and anti-inflammatory mechanisms in health and disease, there is growing interest in identifying natural dietary sources that can effectively modulate these pathways, particularly alternative milk sources that have demonstrated promising bioactive properties. In response to this emerging research priority, this review comprehensively examines the current scientific understanding of the antioxidant and anti-inflammatory bioactivities of camel and donkey milk, specifically exploring their bioactive constituents, mechanisms of action, and potential therapeutic applications. Furthermore, by synthesizing findings from biochemical analyses, in vitro studies, animal models, and the limited but growing body of human trials, this review aims to provide a holistic perspective on the unique health-promoting properties of these alternative milk sources and their potential integration into dietary strategies for health maintenance and disease prevention.

## 2. Methodology for Literature Search

This comprehensive narrative review synthesizes current scientific evidence on the antioxidant and anti-inflammatory potential of camel and donkey milk, conducted following established guidelines for narrative reviews to provide a broad overview of the current state of knowledge while identifying research gaps and future directions. A systematic literature search was conducted across multiple electronic databases, including PubMed/MEDLINE, Web of Science, Scopus, ScienceDirect, and Google Scholar, primarily focused on peer-reviewed articles published between 2017 and May 2025, with additional inclusion of selected seminal studies dating back to 2009 to provide essential foundational context and historical perspective. The search strategy employed the following search terms in various combinations: (“camel milk” OR “donkey milk” OR “non-bovine milk” OR “alternative milk”) and (“antioxidant” OR “anti-inflammatory” OR “bioactive compound”) and (“functional food” OR “health benefit” OR “therapeutic potential”), along with additional terms including “lactoferrin”, “lysozyme”, “immunomodulatory”, “Nrf2”, “NF-κB”, and “cytokine”, and “factors affecting donkey and camel milk composition”.

Only articles published in Science Citation Index (SCI)-indexed international journals in English were considered for inclusion in this review. Studies were selected based on their direct relevance to the antioxidant and anti-inflammatory properties of non-bovine milk sources, with particular emphasis on bioactive compounds and their potential health applications.

Several types of publications were excluded from this review. We excluded book chapters, conference proceedings, unpublished data, and preprint articles. Articles published in non-SCI-indexed journals or languages other than English were also excluded. Additionally, studies focusing solely on processing or technological aspects without bioactivity data were excluded to ensure the inclusion of high-quality, peer-reviewed scientific evidence.

## 3. Comparison of Milk Composition Obtained from Different Non-Bovine Sources

The comprehensive analysis of milk composition across various species (donkey, camel, cow, buffalo, sheep, goat, and human) and their related references have been provided in Table 1. The significant compositional variations observed between these milk sources have profound implications for their functional, nutritional, and technological properties.

### 3.1. pH and Alkalinity Effects

The notably alkaline pH values of human milk (7.0–7.5) and donkey milk (7.0–7.2) compared with other sources [36,37,38] significantly impact their digestibility and antimicrobial properties. This alkaline nature, attributed to lower casein and phosphate content, enhances the survival of beneficial bacteria in the gastrointestinal tract and provides better buffering capacity against gastric acid. The reduced acidity also contributes to improved palatability and reduced protein coagulation during digestion, making these kinds of milks particularly suitable for infant nutrition and individuals with sensitive digestive systems.

### 3.2. Lactose Content and Digestive Implications

The exceptionally high lactose content in human milk (6.69%) and donkey milk (6.2%) compared with camel (4.1%), sheep (4.8%), and goat milk (4.3%) serves multiple functional purposes. Higher lactose levels promote the growth of beneficial Bifidobacterium and Lactobacillus in the intestinal tract, enhance calcium absorption, and provide sustained energy release. This makes donkey milk particularly valuable as a human milk substitute, while the lower lactose content in camel and goat milk offers advantages for lactose-intolerant individuals.

### 3.3. Protein Composition and Functional Properties

The dramatic variation in protein content—from sheep milk’s highest concentration (59.4 g/L) to human milk’s lowest (9–17 g/L)—directly influences the nutritional density and processing characteristics of each milk type. The low casein content in human milk (5.6 g/L) and donkey milk (6.6 g/L) results in softer, more digestible curd formation, reducing digestive stress and improving nutrient absorption. Conversely, sheep milk’s high protein content makes it ideal for cheese production but may require dilution for direct consumption by infants. The elevated whey protein levels in sheep milk (10.76 g/L) and human milk (8 g/L) provide superior amino acid profiles and enhanced immunological benefits due to their bioactive components.

### 3.4. Bioactive Proteins and Health Benefits

The remarkable concentration of lactoferrin in human milk (1.44–4.91 mg/mL) and exceptional lysozyme content in donkey milk (1.00 mg/mL) confer significant antimicrobial and immunomodulatory properties. These bioactive proteins explain the superior infection resistance observed in breastfed infants and suggest therapeutic potential for donkey milk in immunocompromised individuals. The high lysozyme content in donkey milk particularly enhances its natural preservation properties and antimicrobial efficacy.

### 3.5. Fat Content and Fatty Acid Profiles

The wide range in fat percentages—from sheep milk’s richest content (7.0%) to donkey milk’s leanest (0.3–1.8%)—affects both nutritional density and processing suitability. Donkey milk’s high concentration of short-chain fatty acids and omega-3 fatty acids (9.45–9.64%) provides enhanced metabolic benefits and anti-inflammatory properties, while its low total fat content makes it suitable for individuals requiring low-fat diets. Human milk’s elevated monounsaturated fatty acid content (44.30–45.11%) supports optimal brain development and cardiovascular health, explaining its irreplaceable role in infant nutrition.

### 3.6. Vitamin and Mineral Functionality

The superior vitamin C content in sheep milk (41.6 mg/L) enhances its antioxidant capacity and iron absorption properties, while the exceptional vitamin E levels in human milk (3.0–8.0 mg/L) provide crucial membrane protection during rapid infant growth. The optimal calcium/phosphorus ratios in donkey milk (1.72) and human milk (1.7) ensure superior bone mineralization compared with other sources, making these milks particularly valuable during periods of skeletal development. Goat milk’s high potassium content (1810 mg/L) and sheep milk’s elevated zinc levels (5.2–7.47 mg/L) contribute to enhanced cardiovascular function and immune system support, respectively.

These compositional differences collectively determine each milk’s suitability for specific applications: donkey milk emerges as the closest alternative to human milk for infant nutrition, sheep milk excels in cheese production and nutritional density, while goat and camel milk offer advantages for individuals with specific dietary requirements or intolerances.

**Table 1:** Differences in the physicochemical composition of donkey, camel, cow, buffalo, sheep, goat, and human milk.

Items		Donkey	Camel	Cow	Buffalo	Sheep	Goat	Human
pH		7.0–7.2	6.2–6.5	6.4–6.8	5.9–6.4	6.6–7.0	6.4–6.7	7.0–7.5
Carbohydrate	Lactose (%)	6.2	4.1	4.6	4.78	4.8	4.3	6.69
Protein, AA and NPN	Protein (g/L)	15.0–18.0	30–39	33.0	49.2	59.4	33.4	9–17
	Casein (g/L)	6.6	22.1–26.0	24.6–28.0	32.0–40.0	41–66	24–28	5.6
	Whey protein (g/L)	7.5	5.9–8.1	5.5–7.0	6.0	10.76	6.14	8
	α_S1_-casein (g/L)	0.2–1.1	5.3	8.0–10.7	8.9	3.5	3.6	0.3–0.8
	β-casein (g/L)	3.9	15.6	9.5	12.6–20.19	5.33	12–14	1.8–4.0
	α-lactalbumin (g/L)	1.8–3.0	0.8–3.5	1.2–1.3	1.4	1.2–2.6	0.7–2.3	1.9–2.6
	Lactoperoxydase	0.1 g/L	0.17 unit/mL	4.5 unit/mL	2.48–3.22 unit/mL	0.32 unit/mL	1.2 unit/mL	0.77 g/L
	Albumin (g/L)	0.4	7–11.9	0.3–0.4	0.29	0.36	0.56	0.48
	Essential AA (g AA/100 g protein)	38.2	38.0	39.2	41.95	44.1	52.3	40.7
	NPN (mg/L)	455.0	402.0	250–300	500	260	490	454
	Lactoferrin (mg/mL)	0.10–0.13	0.02–7.28	0.02–0.50	0.03–3.4	0.07	0.02–0.2	1.44–4.91
	Lysozyme	1.0 mg/mL	0.00015 mg/mL	0.00018 mg/mL	59.86 U × 10^−3^/mL	0.0001 mg/mL	0.00025 mg/mL	0.39 mg/mL
Lipid	Fat (g/100 g)	0.3–1.8	1.8–4.3	3.7	7.45	7.0	4.5	3.5–4.0
	Triglycerides (g/kg fat)	800–850	960	983	980	980	968	970–980
	Cholesterol (mg/L)	9.0–30.0	345.0	256.3	65–179.6	50–100	100–200	34–290
	Phospholipids (mg/L)	42.8–97.1 mg/L	257.0–660.3 mg/L	195.2–413.4 mg/L	3.22 mg/g fat	308.1 mg/L	195.5–202.1 mg/L	98–474 mg/L
	Fatty acid							
	C8:0 (g/kg fat)	24.5–40.3	-	12.0	24.1	26.4	27.3	1.6–2.8
	C10:0 (g/kg fat)	59.9–98.0	3.2	26.0	24.0	31.3	25.3	14.6–25.3
	C 16:0 (g/kg fat)	200–220	359.7	259.0	280.2	231.1	242.0	190–230
	C 18:1 (g/kg fat)	110–280	163.4	276.0	241.0	260.1	230.1	210–360
	C 18:2 n6 (g/kg fat)	90–170	17.3	21.0	20.4	16.1	27.2	123–139
	C18:3 n3 (g/kg fat)	20–140	2.6	7.0	6.8	9.2	5.3	8–11
	SFA % total fatty acids	46.7–67.7	68.13	68.72	69.04	64.23	73.69	39.41–42.24
	MUFA % total fatty acids	15.3–35.0	29.2	27.40	28.46	28.0	20.88	44.30–45.11
	PUFA % total fatty acids	15.2–30.5	2.6	4.05	2.5	4.82	5.43	15.48
	PUFA n3 % total fatty acids	9.45–9.64	2.42	0.8	0.7	1.0	0.47	1.27–2.19
	PUFA n6 % total fatty acids	11.57–13.09	0.27	2.3	1.7	2.97	4.96	11.17–14.1
Vitamin	Vitamin A (mg/L)	0.017	0.10	1.7	0.06	0.38–0.53	0.32–0.40	0.30–0.70
	Vitamin C (mg/L)	12–57	37.4	0.9	25.0	41.6	12.9	38–53
	Vitamin D	2.23 (µg/100 mL)	1–16 (μg/L)	2 (mg/100 g)	2 (mg/100 g)	1.18 (mg/100 g)	1.33 (mg/100 g)	0.06 µg/100 mL
	Vitamin B2 (mg/L)	0.64	0.57	1.7	1.1	3.7	2.1	0.40–0.60
	Vitamin E (mg/L)	0.051	0.56	2.1	1.9	1.97–2.14	1.29	3.0–8.0
Minerals	K (mg/L)	240–747	520–1800	1520	920	1360–1400	1810	530
	Na (mg/L)	100–268	220–690	580	350	440–580	410	180
	Mg (mg/L)	40–83	59–97	200	270	53.9	32	35
	Phosphorus	43.44 (mg/100 g)	0.34–1.00 (g/L)	119 (mg/100 g)	119 (mg/100 g)	124–158 (mg/100 g)	121 (mg/100 g)	137.1 (mg/L)
	Ca/P	1.72	1.45	1.03	1.13	1.57	1.1	1.7
	Zn (mg/L)	1.23–3.19	5.8	5.3	4.1	5.2–7.47	0.56	1–3

Note: AA—Amino Acid; NPN—Nonprotein Nitrogen; SFA—Saturated Fatty Acids; MUFA—Monounsaturated Fatty Acid; PUFA—Polyunsaturated Fatty Acid. The information provided in Table 1 is based on the following published data [3,8,20,39,40,41,42,43,44,45,46,47,48,49,50,51,52,53,54,55,56,57,58,59,60,61,62,63,64,65,66,67,68,69,70].

## 4. The Anti-Inflammatory and Immune-Regulating Effects of Donkey Milk

Donkey milk contains a high proportion of whey proteins, including β-lactoglobulin, α-lactalbumin, serum albumin, lactoferrin, lysozyme, and immunoglobulins [1,71,72,73]. Lysozyme stands out as a particularly abundant component, comprising 13% of total whey proteins at an average concentration of 1.07 g/L [74,75]. This enzyme disrupts the peptidoglycan layer of gram-positive bacteria and shows remarkable activity levels ranging from 1670 to 11,531 U/mL [76,77]. Compared with other milk types, donkey milk’s lysozyme content closely resembles human milk (0.3–1.1 g/L) [78] but far exceeds cow’s milk, which contains negligible amounts. Similarly, while lysozyme activity in donkey milk is substantial, it remains lower than human milk (approximately 39,000 U/mL) but dramatically higher than cow’s milk (0.0292 U/mL).

Research by Mao et al. [79] indicates that the whey protein fractions containing lysozyme are responsible for donkey milk’s immunomodulatory effects. However, the potential contributions of other bioactive components to these immunomodulatory properties require further investigation. Specifically, the roles of α-lactalbumin and lactoferrin in immune modulation remain to be fully determined. α-lactalbumin functions as a multifunctional bioactive protein that regulates immune responses in infants through multiple pathways. These mechanisms include serving as a precursor for antimicrobial peptide formation and modulating inflammatory responses. This whey protein also facilitates the absorption of essential minerals and supports the development of the intestinal barrier function, which is crucial for immune system maturation. Concurrently, milk oligosaccharides function as prebiotic compounds that selectively promote the growth of beneficial gut microbiota, particularly *bifidobacteria* and *lactobacilli*. These complex carbohydrates serve as decoy receptors for pathogenic bacteria, preventing their adhesion to intestinal epithelial cells while simultaneously stimulating the production of immunomodulatory cytokines. Recent investigations have revealed that oligosaccharides significantly enhance immune system development by promoting T-cell differentiation, supporting the maturation of gut-associated lymphoid tissue, and establishing long-term immune memory patterns that extend well beyond the weaning period [79]. Nevertheless, research on oligosaccharides has focused primarily on human milk, with donkey milk receiving considerably less attention in this regard [80]. The medicinal properties and antioxidant characteristics of donkey milk have been documented extensively throughout history, with traditional applications including wound healing and the treatment of various conditions such as joint pain, asthma, bronchitis, and gastritis [78,81].

Donkey milk contains several bioactive components with significant anti-inflammatory properties. These components suppress the nuclear factor kappa-light-chain-enhancer of activated B cells (NF-κB) pathway, thereby reducing inflammatory responses [4,6,82]. Lactoferrin represents one of the most prominent bioactive proteins in donkey milk. This protein exerts its anti-inflammatory effects by inhibiting the inhibitor of kappa-B kinase (IκB) stimulation [83,84]. Through this mechanism, lactoferrin prevents the phosphorylation and subsequent degradation of IκB proteins. This mechanism maintains NF-κB in its inactive cytoplasmic state, blocking its nuclear translocation and transcriptional activity of pro-inflammatory genes. Additionally, oligosaccharides and bioactive peptides derived from donkey milk proteins directly interfere with the binding of NF-κB to its DNA recognition sites, further suppressing inflammatory responses. The high concentration of polyunsaturated fatty acids, particularly omega-3s, disrupts lipid raft formation in cell membranes, which impairs the assembly of the NF-κB activation complex [85,86].

Animal model studies have demonstrated the immunomodulatory properties of donkey milk, revealing its ability to regulate immune system function through diverse mechanistic pathways [79,87,88]. These investigations have shown that donkey milk administration produces immunoregulatory effects, including the upregulation of critical pro-inflammatory cytokines that facilitate immune system regulation and coordinate adaptive responses [79,87,88]. Specifically, these studies have documented significant increases in the production of interleukin-1 (IL-1), interleukin-6 (IL-6), and tumor necrosis factor-α (TNF-α), which represent critical mediators of the innate immune response and inflammatory cascade [87]. Complementing these pro-inflammatory effects, donkey milk simultaneously demonstrates anti-inflammatory properties through the induction of interleukin-10 (IL-10) release, a pivotal regulatory cytokine responsible for modulating excessive inflammatory responses and maintaining immune homeostasis [88]. This dual immunomodulatory capacity suggests that donkey milk possesses sophisticated regulatory mechanisms that can both activate and balance immune responses as physiologically required. The bioactive whey protein fraction of donkey milk has been identified as a primary mediator of these immunomodulatory effects. In vitro studies utilizing mouse spleen cell cultures have demonstrated that donkey milk whey proteins possess the capacity to stimulate the production of specific immunoregulatory cytokines, including interferon gamma (IFN-γ), a crucial mediator of cellular immunity and macrophage regulation [79]. These findings have been corroborated by in vivo experiments wherein mice subjected to intragastric administration of donkey milk exhibited significantly elevated levels of IL-6 and TNF-α, results that demonstrate remarkable consistency with previously published investigations [79]. The observed cytokine profile enhancement provides compelling evidence for the augmentation of immune system capacity in experimental animals following donkey milk intervention, with these immunostimulatory effects being predominantly attributed to the unique composition and bioactivity of donkey milk whey proteins.

The molecular basis of donkey milk’s immunomodulatory activity can be attributed to several specific bioactive components within the whey protein fraction. Key immunologically active molecules, including fibrinogen β chain, annexin A1, and toll-like receptors (TLRs), have been identified as integral components of donkey milk whey proteins, with each playing distinct and critical roles in the initial recognition and response phases of innate immunity [89]. These proteins function as essential mediators in the first line of immunological defense, facilitating pathogen recognition and initiating appropriate immune responses. Among the bioactive components, lactoferrin represents a particularly significant immunomodulatory factor, with donkey milk containing exceptionally high concentrations of this multifunctional glycoprotein [90]. Lactoferrin exhibits extensive biological activities encompassing antimicrobial, anti-inflammatory, and immunoregulatory properties [91] while demonstrating excellent bioavailability due to its resistance to degradation by gastric acid and susceptibility to processing by duodenal digestive enzymes [92]. These pharmacokinetic characteristics contribute substantially to the sustained immunoregulatory effects observed following donkey milk consumption.

The immunoglobulin content of donkey milk represents another crucial component contributing to its immunomodulatory capacity. Donkey milk contains substantial concentrations of various immunoglobulins [93], which provide passive immunity transfer and contribute to the overall enhancement of immune system function. The presence of these antibodies may facilitate improved pathogen recognition and clearance, thereby supporting both innate and adaptive immune responses. The thymus gland, recognized as the primary organ responsible for T-cell maturation and adaptive immune system development, serves as an important indicator of immune system status and functionality. Experimental evidence has demonstrated that mice receiving intragastric donkey milk administration exhibit significantly increased thymus index values, providing quantitative evidence for the enhancement of immune organ function and capacity [93]. This finding represents additional confirmation that donkey milk consumption results in measurable improvements in immune system development and function. Clinical validation of these immunomodulatory effects has been demonstrated through multiple animal disease models, where donkey milk administration has proven effective in ameliorating inflammatory pathologies. Therapeutic efficacy has been documented in Balb/c mice with experimentally induced colitis [87], C57BL/6 mice with ileitis [94], and Wistar rats with diabetes-associated inflammatory complications [95].

Donkey milk demonstrates significant immunomodulatory properties that have been extensively characterized through both in vitro experimental systems and validated through rigorous randomized controlled trials conducted in animal models and human subjects [79,87,88,96]. The immunological activity of donkey milk is primarily attributed to its capacity to modulate cytokine production, which represents a fundamental mechanism underlying immune system regulation and inflammatory response coordination during pathogenic challenges. Comprehensive in vitro investigations have established that donkey milk possesses the ability to stimulate the release of key pro-inflammatory cytokines that are essential components of the innate immune response system [79,87,88,97]. Specifically, donkey milk administration has been demonstrated to enhance the production of IL-1, IL-6, and TNF-α, which collectively function as critical mediators in the initiation and maintenance of local acute inflammatory responses necessary for effective pathogen recognition and elimination. These cytokines play pivotal roles in orchestrating the recruitment and induction of immune effector cells, thereby enhancing the host’s capacity to mount appropriate defensive responses against infectious agents. However, the immunomodulatory effects of donkey milk exhibit notable context-dependent characteristics, as evidenced by the findings of Jiang et al. [87], who reported significant inhibition of TNF-α production in murine models of inflammatory bowel disease (IBD). This observation suggests that donkey milk may possess adaptive immunoregulatory properties that can either promote or suppress inflammatory responses depending on the underlying pathophysiological conditions and the specific immune microenvironment present at the time of administration. Further mechanistic studies have revealed that the high-molecular-weight whey protein fraction of donkey milk, specifically components exceeding 10 kDa, demonstrates potent immunostimulatory activity through its capacity to activate murine splenocytes [79]. This stimulation results in the enhanced production of specialized immune regulatory cytokines, including IL-2 and IFN-γ, which are essential for T-cell proliferation, differentiation, and the coordination of adaptive immune responses. The stimulation of these particular cytokines indicates that donkey milk may possess the ability to enhance cell-mediated immunity and promote the development of immunological memory.

Concurrently, donkey milk has been shown to induce the production of IL-10, a potent anti-inflammatory cytokine that serves multiple regulatory functions, including the suppression of excessive inflammatory reactions, facilitation of pathogen clearance mechanisms, and mitigation of infection-associated tissue damage [79]. The dual capacity to stimulate both pro-inflammatory and anti-inflammatory mediators suggests that donkey milk may function as a sophisticated immunomodulator capable of maintaining homeostatic balance within the immune system. This balanced immunomodulatory profile was further corroborated by Jiang et al. [87], who documented significant inhibition of inflammatory mediator expression, particularly IL-13 and TNF-α, in murine models of inflammatory diseases, thereby demonstrating the therapeutic potential of donkey milk in managing inflammatory pathologies through its anti-inflammatory mechanisms.

The anti-inflammatory properties of donkey milk are primarily attributed to several key components, including IFN-γ, lactoferrin, lysozyme, transforming growth factor beta (TGF-β), and lactic acid bacteria [94]. TGF-β is considered a potent secretory immunosuppressive cytokine that reduces inflammation by inhibiting the activation of immune cells like T cells and macrophages while promoting the development of regulatory T cells that actively suppress inflammatory responses. It also inhibits the production of pro-inflammatory cytokines such as TNF-α and IL-1β and simultaneously promotes tissue repair and healing, making it a key component in donkey milk’s anti-inflammatory properties. Contemporary scientific investigations have increasingly directed attention toward the comprehensive identification and precise quantification of naturally occurring bioactive peptides within both colostral and mature donkey milk samples, with particular emphasis on the characterization of peptides exhibiting anti-inflammatory properties and those demonstrating angiotensin-converting enzyme (ACE)-inhibitory activity [98]. In addition to direct cytokine modulation, donkey milk also influences the gut microbiota, which plays a key role in immune system function. Consistently, a study documented that intragastric donkey milk administration (10 mL per kg body weight) increased inflammatory markers (TNF-α and IL-6) in mouse liver tissue and altered both gut bacterial composition and blood metabolite levels. This treatment altered the metabolic profile, resulting in the identification of 145 distinct metabolites, notably nicotinamide, L-valine, and β-estradiol, which modulated amino acid metabolism and fatty acid biosynthesis pathways. Concurrent gut microbiome analysis revealed an increased abundance of *Lactobacillus* species, with specific bacterial taxa (*Falsiroseomonas* and *Salipiger*) demonstrating positive correlations with coenzyme Q biosynthesis, potentially contributing to enhanced immune function [99].

Recent scientific investigations have increasingly focused on the anti-inflammatory and immunomodulatory properties of donkey milk, with a substantial number of studies emerging in this area (Table 2). The foundational work conducted by Kocyigit et al. [7] established the preliminary framework for understanding the anti-inflammatory potential of equid milk, thereby creating the scientific foundation for subsequent investigative endeavors in this specialized field of research. Advancing beyond these initial observations, Li Y et al. [20] provided compelling evidence that corroborated and expanded upon the established anti-inflammatory paradigm. Their comprehensive analysis demonstrated that the synergistic anti-inflammatory and antioxidative properties inherent in donkey milk constitute fundamental mechanisms underlying its significant health-promoting and regulatory effects on human physiological systems.

In a particularly noteworthy investigation, Taghiloo et al. [100] conducted an extensive analysis to elucidate the complex immunomodulatory mechanisms through which donkey milk influences immune cell functionality. Their findings revealed that systematic donkey milk treatment resulted in significant upregulation of specific cytokines, including IL-8 and IL-6, within peripheral blood mononuclear cells (PBMCs). The elevation of IL-8 is particularly significant given its established role as a potent chemotactic factor responsible for neutrophil recruitment and activation. Similarly, IL-6 functions as a pleiotropic cytokine that mediates acute-phase responses and exhibits cytoprotective properties. Furthermore, donkey milk administration resulted in moderate enhancement of TNF-α production from PBMCs, indicating a measured pro-inflammatory response that maintains homeostatic balance without triggering excessive inflammatory activation. Notably, the stimulation of normal human PBMCs with donkey milk consistently induced the release of IL-10 into culture supernatants, thereby highlighting donkey milk’s capacity to orchestrate immune response modulation and maintain immunological homeostasis [100]. These specific cytokine mediators are recognized as essential regulators in the complex processes of immune cell maturation, differentiation, and functional regulation, ultimately serving to enhance and optimize the host’s innate and adaptive defense mechanisms [101].

Emerging evidence from immunological investigations has demonstrated that donkey milk possesses significant bioactive properties capable of modulating host immune responses. Specifically, previous research has established that donkey milk components can effectively stimulate nitric oxide (NO) biosynthesis in PBMCs, a critical mechanism underlying innate immune activation. Moreover, these bioactive constituents facilitate the enhancement of adaptive immune responses through the orchestrated production of immunoregulatory cytokines, thereby contributing to overall immune system homeostasis [101,102]. The therapeutic efficacy of donkey milk has been extensively validated through controlled animal model investigations, which have revealed multifaceted beneficial effects on gastrointestinal health and immune function. In experimental studies utilizing murine models of inflammatory bowel disease, specifically ileitis-induced pathology, donkey milk administration demonstrated pronounced anti-inflammatory properties characterized by significant attenuation of intestinal inflammation. These therapeutic effects were mechanistically attributed to the restoration of antimicrobial peptide homeostasis, including the normalization of lysozyme and α-defensin expression levels within specialized Paneth cells of the intestinal crypts. Concurrently, donkey milk treatment effectively ameliorated intestinal dysbiosis, a hallmark pathological feature associated with chronic ileitis, thereby promoting the restoration of beneficial microbial communities [94]. Furthermore, additional preclinical evidence has substantiated the protective effects of donkey milk on intestinal barrier integrity and function. In experimental paradigms employing water-avoidance stress-induced gastrointestinal dysfunction in rodent models, donkey milk supplementation demonstrated significant preservation of gut barrier function, as evidenced by improved intestinal permeability parameters and enhanced epithelial barrier maintenance. These findings collectively underscore the considerable therapeutic potential of donkey milk as a functional food intervention for gastrointestinal disorders and stress-related intestinal dysfunction [103].

Human studies investigating the immunomodulatory effects of donkey milk remain limited. The primary clinical study involved elderly subjects, specifically 14 healthy aged individuals ranging from 72 to 97 years, in which donkey milk was compared with goat milk [76]. These researchers observed that the administration of 200 mL of donkey milk daily for one month acts as an enhancer of acute phase response in humans. Based on these findings, daily donkey milk consumption may be recommended in the diet of immunocompromised elderly patients, suggesting its potential therapeutic application in vulnerable populations.

## 5. Antioxidant Potential of Donkey Milk

Donkey milk demonstrates exceptional antioxidant capacity attributed to its rich composition of bioactive peptides, ascorbic acid (vitamin C), polyunsaturated fatty acids, and a comprehensive profile of essential amino acids [6,104,105]. These bioactive compounds collectively contribute to the milk’s potent free radical scavenging capabilities and cellular protective mechanisms against oxidative stress-induced damage. The antioxidant efficacy of donkey milk has been rigorously evaluated through methodologically sound, double-blind, randomized controlled trials utilizing established animal models [95,97,106]. These comprehensive investigations have consistently demonstrated significant enhancement of endogenous antioxidant defense systems and upregulation of phase II detoxifying enzyme activities in experimental subjects receiving donkey milk supplementation [95,97,106]. Consistently, Li and colleagues [83] conducted pivotal research demonstrating that donkey milk consumption significantly enhanced SOD enzymatic activity in plasma samples obtained from diabetic rat models compared with control groups receiving standard treatment protocols. The superoxide dismutase enzyme represents a critical first-line defense mechanism, catalyzing the dismutation reaction of highly reactive superoxide anion radicals (O_2_^−^) into molecular oxygen (O_2_) and hydrogen peroxide (H_2_O_2_), thereby preventing oxidative damage to cellular components. Furthermore, the same research group [95] provided compelling evidence that total antioxidant capacity was substantially improved in diabetic animals receiving donkey milk therapy compared with untreated control groups, with measured values approaching those observed in healthy, non-diabetic control animals. This finding suggests that donkey milk supplementation may effectively restore antioxidant homeostasis in pathological conditions characterized by oxidative stress.

Comprehensive murine model investigations have revealed additional significant improvements in critical oxidative stress biomarkers and hepatic detoxification mechanisms. Researchers documented substantial improvements in the glutathione-to-glutathione disulfide (GSH/GSSG) ratio within hepatic tissue, which serves as a fundamental indicator of cellular redox status and oxidative stress burden [97,106]. Concomitantly, enhanced enzymatic activities of crucial phase II detoxifying enzymes, including glutathione-S-transferase (GST) and NAD(P)H:quinone oxidoreductase (NQO1), were observed in liver tissue samples [97,106]. These findings collectively demonstrate that regular donkey milk consumption significantly enhances the organism’s intrinsic antioxidant defense mechanisms and provides robust cellular protection against oxidative damage.

Direct quantitative assessment of antioxidant activities in both raw donkey milk and fermented donkey milk products (kefir) utilizing standardized 2,2′-Azino-bis(3-ethylbenzothiazoline-6-sulphonic acid) (ABTS) and 2,2-diphenyl-1-picrylhydrazyl (DPPH) radical scavenging assays revealed significantly higher antioxidant capacity in fermented products compared with unprocessed milk [105]. Notably, these antioxidant activities demonstrated further enhancement following in vitro simulated gastrointestinal digestion protocols, suggesting improved bioavailability and biological activity of antioxidant compounds post-consumption [105]. The enhanced antioxidant activity observed in fermented donkey milk has been specifically attributed to the metabolic activities of particular probiotic bacterial strains, most notably *Enterococcus faecium DM33*, as fermented milk products containing this specific microorganism demonstrated the most pronounced antioxidant activity among tested formulations [105]. The elevated antioxidant properties are hypothesized to result from the enzymatic liberation of bioactive peptides through proteolytic hydrolysis of milk proteins during bacterial fermentation processes and subsequent gastrointestinal digestion.

A comprehensive peptidomic characterization conducted by Piovesana et al. [107] successfully identified and characterized 1330 distinct peptide sequences from commercially available donkey milk samples. The majority of these bioactive peptides were determined to originate from β-casein, αS1-casein, and serum amyloid A protein through proteolytic cleavage mechanisms. Additionally, β-lactoglobulin I and lactoferrin were identified as significant sources of bioactive milk peptides, while α-lactalbumin and lysozyme demonstrated remarkable resistance to gastrointestinal enzymatic degradation [108]. When subjected to rigorous in vitro bioassay protocols, donkey milk-derived peptide fractions consistently exhibited significant and dose-dependent antioxidant activities, providing substantial evidence supporting the therapeutic potential of these naturally occurring bioactive compounds [108,109].

Numerous peptides identified in donkey milk possess characteristic structural features consistent with ACE-inhibitory peptides, demonstrating significant potential for reducing ACE enzymatic activity and subsequently modulating cardiovascular function [108]. In vitro bioassay protocols have consistently confirmed the presence of potent angiotensin-converting enzyme inhibitory activities in various donkey milk peptide fractions [107,110]. While the potentially bioactive peptides identified in donkey milk by Zenezini Chiozzi and colleagues [110] did not exhibit complete sequence homology with previously characterized bioactive peptides, several candidate sequences displayed notable structural similarities to established bioactive compounds. Notably, a putative ACE-inhibitory peptide (MPFLKSPIVPF) showed significant sequence similarity and identical peptide length compared with a validated antihypertensive peptide (MPFPKYPVQPF) previously isolated and characterized from aged Gouda cheese.

The cardiovascular protective mechanisms mediated by milk-derived bioactive peptides involve the coordinated modulation of the renin-angiotensin-aldosterone system through potential inhibition of angiotensin II formation and concurrent enhancement of bradykinin levels. These complementary physiological actions function synergistically, as angiotensin II possesses potent vasoconstrictor properties while bradykinin demonstrates significant vasodilatory effects, collectively resulting in blood pressure reduction and improved cardiovascular function. Supporting this mechanistic hypothesis, fermented donkey milk preparations containing *Lactobacillus casei* DM214 demonstrated significant ACE-inhibitory activity in controlled in vitro experimental systems [105]. Furthermore, in vitro experimental protocols have revealed that donkey milk treatment stimulates NO release from PBMCs. Given that nitric oxide functions as a potent endothelium-derived vasodilator and plays a crucial role in vascular homeostasis, researchers have proposed a significant potential role for donkey milk in atherosclerosis prevention and cardiovascular disease management [96].

Contemporary experimental research has elucidated novel therapeutic applications of donkey milk, extending beyond its established cardiovascular benefits to include gastroprotective properties. In controlled preclinical studies, prophylactic administration of donkey milk for 10 consecutive days prior to ethanol challenge demonstrated significant gastroprotective efficacy, as evidenced by marked attenuation of ethanol-induced gastric mucosal lesions in murine experimental models [111]. The gastroprotective mechanism appears to involve modulation of oxidative stress pathways, as evidenced by a significant reduction in malondialdehyde (MDA) concentrations—a key biomarker of lipid peroxidation—coupled with enhanced glutathione (GSH) expression, indicating upregulation of endogenous antioxidant defense systems in gastric tissue [111]. These findings suggest that donkey milk’s gastroprotective efficacy may be mediated through its ability to preserve gastric mucosal integrity via antioxidant-dependent mechanisms, warranting further mechanistic elucidation and potential clinical translation.

Corroborating evidence for the proposed antioxidant mechanisms derives from well-controlled animal studies conducted by Trinchese et al. [97], who observed significant reductions in hydrogen peroxide concentrations in experimental rats receiving donkey milk and human milk supplementation. The research team documented inhibition of succinate dehydrogenase enzymatic activity concurrent with enhanced superoxide dismutase activity, accompanied by measurable improvement in cellular redox status as quantified by the GSH/GSSG ratio. The investigators attributed these beneficial physiological outcomes to the activation of the Nrf2-ARE signaling pathway [97]. Consistent findings were reported by Lionetti et al. [112], who discovered that experimental rats receiving donkey milk supplementation exhibited significantly increased hepatic glutathione concentrations while demonstrating enhanced enzymatic activities of quinone oxidoreductase 1 (NQO1) and glutathione S-transferase (GST) within mitochondrial compartments. These experimental observations align with established molecular understanding that under conditions of mild oxidative stress, the Nrf2 transcription factor is released from its cytoplasmic inhibitor Kelch-like ECH-associated protein 1 (Keap1), subsequently triggering transcriptional activation of genes encoding protective enzymes, including NQO1 and GST [113]. However, it is important to acknowledge that donkey milk composition, antioxidant status, anti-inflammatory properties, and immune responses have been demonstrated to be significantly influenced by various environmental and physiological factors, including dietary composition, lactation stage, and breed-specific genetic variations [114,115,116,117,118,119,120]. These variables must be carefully considered when evaluating the therapeutic potential and standardizing donkey milk products for clinical applications. The comprehensive biological activities and therapeutic properties of donkey milk have been systematically documented and are summarized in Table 2, providing a consolidated reference for the multifaceted health benefits associated with this unique dairy product.

## 6. Anti-Inflammatory and Antioxidant Potential of Camel Milk

Camel milk contains numerous bioactive compounds, including lactoferrin, α-lactalbumin, and vitamin C, which contribute significantly to its antioxidant capacity [24,129,130]. The multifaceted therapeutic properties of camel milk, encompassing potent antioxidant, antimicrobial, and anti-inflammatory activities, have been comprehensively characterized and validated through extensive research investigations [131,132]. Parkinson’s disease (PD) pathogenesis is characterized by a complex interplay of deleterious cellular processes, including excessive oxidative stress generation, chronic neuroinflammation, mitochondrial dysfunction, and dysregulated apoptotic pathways, which collectively contribute to progressive neurodegeneration. In this pathological context, the camel milk α-lactalbumin and oleic acid (CLOA) complex has emerged as a promising neuroprotective agent through its sophisticated molecular mechanism of action. Specifically, the CLOA complex exerts its therapeutic effects via the selective activation of silent information regulatory protein 1 (SIRT1), a critical NAD+-dependent deacetylase that serves as a master regulator of cellular stress responses and metabolic homeostasis [133]. The SIRT1-mediated neuroprotective cascade initiated by CLOA involves the effective attenuation of oxidative stress burden and the maintenance of hypoxia-inducible factor-1α (HIF-1α) in its functionally active deacetylated state, thereby preserving cellular adaptation mechanisms under stress conditions [133]. Subsequently, activated SIRT1 orchestrates the upregulation of key transcription factors, including forkhead box O3a (FOXO3a) and heat shock factor 1 (HSF-1), which function as critical mediators of cellular survival pathways. This transcriptional activation results in a significant reduction in apoptotic cell death and maintenance of protein homeostasis through enhanced cellular stress response mechanisms. Furthermore, the therapeutic efficacy of CLOA is amplified by its anti-inflammatory properties, as elevated SIRT1 expression demonstrates a strong inverse correlation with the production of pro-inflammatory cytokines, including TNF-α, IL-6, and IL-8, thereby effectively inhibiting neuroinflammatory cascades that contribute to neuronal damage and disease progression [133].

Camel milk exhibits substantial antioxidant properties primarily attributed to its bioactive constituents, which effectively inhibit the generation of reactive oxygen species (ROS) and consequently reduce oxidative stress. Evidence indicates that camel milk enhances endogenous antioxidative defense mechanisms, improves insulin sensitivity, and supports hepatic and renal function [24]. In streptozotocin (STZ)-induced diabetic rabbit models, camel milk administration significantly enhanced the enzymatic activity of SOD, GPx, and CAT while concomitantly reducing MDA levels [134,135].

Camel milk demonstrates potent anti-inflammatory activity by suppressing key inflammatory mediators, including TNF-α, IL-6, and IL-1β, in lipopolysaccharide (LPS)-induced inflammation models using RAW 264.7 macrophage cell lines [136,137,138,139]. Consistently, Shukla et al. [140] demonstrated that *Lacticaseibacillus paracasei* (M11)-fermented camel milk effectively suppressed LPS-induced pro-inflammatory cytokines and their associated mediators, including NO, TNF-α, IL-6, and IL-1β in RAW 264.7 cells. Further investigation has revealed the anti-inflammatory and antioxidative properties of bioactive peptides isolated from camel milk fermented with *Lacticaseibacillus casei* NK9, which similarly suppressed LPS-induced inflammation in RAW 264.7 cells. Molecular docking analyses predicted that specific peptides (LLNEK and IYTFPQPQSL) could inhibit neutrophil myeloperoxidase (nMPO) activity through interactions with key residues within the human myeloperoxidase (hMPO) active site [141]. Emerging evidence demonstrates that camel milk confers cytoprotective effects through diverse molecular mechanisms across multiple murine toxicity models, suggesting broad therapeutic potential for various pathological conditions. Consistently, Ming et al. [142] elucidated the hepatoprotective mechanisms of camel milk in alcohol-induced liver injury, demonstrating amelioration through microbiome modulation and immunomodulatory pathways. The intervention resulted in significant downregulation of pro-inflammatory cytokines, including TNF-α, IL-6, and IL-1β, while concurrently upregulating the IL-10 expression. Additionally, camel milk enhanced endogenous antioxidant defense systems, as evidenced by elevated SOD and GSH levels, alongside reduced MDA expression [142].

**Figure 2:**
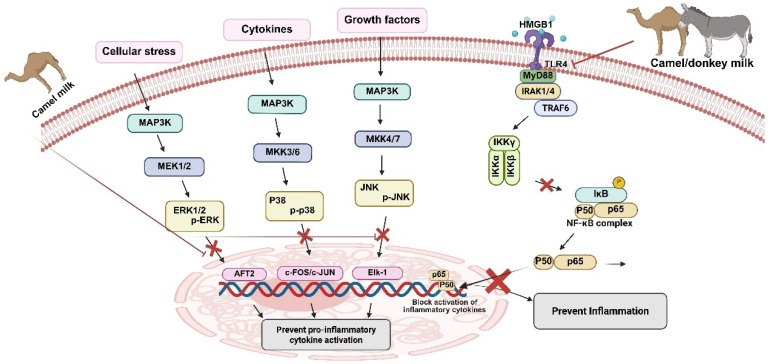
The molecular mechanism of donkey and camel milk to combat inflammation. The figure illustrates that camel and donkey milk work through multiple mechanisms to inhibit the NF-κB inflammatory pathway, including regulation of the HMGB1/TLR4/NF-κB/MyD88 signaling pathway and downregulation of NF-κB pathway activation by decreasing protein expression of activated NF-κBp65, p-NF-κBp65, and p-IκBα proteins. In addition, the figure demonstrates that camel milk is particularly effective as an anti-inflammatory agent—by targeting the phosphorylation step that is essential for MAPK activation, it can simultaneously shut down multiple pro-inflammatory pathways (ERK, p38, and JNK) that would otherwise lead to production of inflammatory mediators’ land matrix metalloproteinases.

Corroborating these findings, Zhu et al. [143] reported that camel milk administration significantly attenuated alcohol-induced elevations in alanine aminotransferase (ALT), aspartate aminotransferase (AST), triglycerides (TG), MDA, and pro-inflammatory cytokines. The hepatoprotective effects were mediated through enhanced oxidative stress control via upregulation of CAT, GPx, and GSH levels, concomitant with reduced lipid accumulation and improved gut microbiota composition in experimental rats [143]. In nephrotoxicity models, camel milk (10 mL/kg) demonstrated significant renoprotective effects against cyclosporine A-induced renal injury [25]. Treatment resulted in substantial reductions in serum creatinine, blood urea nitrogen, and neutrophil gelatinase-associated lipocalin (NGAL) concentrations. The nephroprotective mechanisms involved activation of the Nrf2/heme oxygenase-1 (HO-1) signaling pathway, resulting in enhanced expression of antioxidant enzymes, including GSH, GPx, and SOD. Furthermore, camel milk modulated apoptotic signaling cascades, characterized by increased proliferating cell nuclear antigen (PCNA) expression and decreased pro-apoptotic marker expression, specifically Bcl-2-associated X protein (Bax), poly (ADP-ribose) polymerase (PARP), and caspase-3 [25]. The nephroprotective effects were further mediated through activation of the phosphatidylinositol 3-kinase (PI3K)/protein kinase B (Akt)/endothelial nitric oxide synthase (eNOS)/NO signaling pathway [25]. This PI3K/Akt/eNOS/NO signaling axis appears to be central to camel milk’s nephroprotective mechanisms [27,144,145]. In brief, they demonstrated that camel milk administration (10 mL/kg, twice daily) effectively prevented fluorouracil and methotrexate (20 mg/kg)-induced nephrotoxicity. These chemotherapeutic agents typically compromise renal function through the depletion of antioxidant enzyme reserves (SOD, CAT, GPx) while simultaneously upregulating pro-apoptotic proteins (Bax, p53) and downregulating anti-apoptotic factors (Bcl-2, PCNA) [27,144]. Camel milk administration counteracted these pathological alterations through PI3K/Akt/eNOS pathway activation, as evidenced by enhanced PI3K p110, phospho-Akt, and phospho-eNOS expression levels [27,144]. Consistently, a study examined the effects of oral camel milk administration (10 mL/kg/day via gavage for 21 days) on cyclosporine-induced renal injury [26]. It was revealed that camel milk significantly ameliorated renal dysfunction by reducing serum creatinine, blood urea nitrogen (BUN), and kidney injury molecule-1 (KIM-1) levels while exerting potent anti-inflammatory effects through suppression of pro-inflammatory cytokines (MCP-1, TNF-α, IL-1β, and IL-18), downregulation of matrix metalloproteinases (MMP-2 and MMP-9), and enhancement of anti-inflammatory IL-10 production. Mechanistically, camel milk inhibited both p38/ERK/JNK MAPK and NF-κB signaling pathways by reducing phosphorylation of p38 MAPK, JNK1/2, and ERK1/2 and downregulating NF-κBp65, p-NF-κBp65, and p-IκBα protein expression (Figure 2). Additionally, camel milk effectively countered oxidative stress by reducing myeloperoxidase activity, improving the GSH/GSSG ratio, and enhancing total antioxidant capacity [26]. Furthermore, a study observed an analogous protective mechanism in a 2,4,6-trinitrobenzene sulfonic acid (TNBS)-induced experimental colitis model, where camel milk administration resulted in a significant reduction in pro-inflammatory cytokine levels, suggesting conservation of cytoprotective signaling pathways across diverse pathological conditions [145]. These investigations collectively demonstrate that camel milk exerts multimodal cytoprotective effects through four principal mechanisms: (1) modulation of inflammatory signaling cascades with selective cytokine regulation, (2) enhancement of cellular antioxidant defense systems through upregulation of enzymatic and non-enzymatic antioxidants, (3) regulation of apoptotic signaling pathways favoring cell survival, and (4) activation of the PI3K/Akt/eNOS survival pathway promoting cellular homeostasis. The convergent mechanistic pathways observed across hepatotoxicity, nephrotoxicity, and inflammatory models suggest that camel milk possesses broad cytoprotective potential. Further elucidation of these molecular mechanisms may facilitate the development of novel therapeutic interventions for pathological conditions characterized by inflammation, oxidative stress, and dysregulated apoptotic signaling.

A controlled clinical investigation evaluated the antidiabetic efficacy of camel milk-derived lactoferrin supplementation (250 mg/day) in sixty obese pediatric patients diagnosed with type 2 diabetes mellitus, compared with standard therapeutic regimens alone and fifty healthy control subjects over a three-month intervention period [146]. Lactoferrin supplementation demonstrated significant therapeutic improvements, including substantial reductions in glycated hemoglobin (HbA1c) concentrations, enhanced body mass index (BMI) normalization, and ameliorated lipid metabolic profiles. These clinical improvements were accompanied by upregulated expression of peroxisome proliferator-activated receptor-γ (PPAR-γ) and SIRT-1. The intervention elicited pronounced anti-inflammatory effects, characterized by significant decreases in pro-inflammatory cytokine levels, including IL-1β, IL-6, IL-18, TNF-α, and lipocalin-2, which correlated with reduced NF-κB signaling activation. Enhanced antioxidant capacity was confirmed through elevated SOD enzymatic activity and Nrf2 expression, establishing the mechanistic involvement of the TLR4-NF-κB-SIRT-1 signaling cascade in lactoferrin’s therapeutic action [146]. Corroborating clinical findings, Alharbi et al. [135] demonstrated that camel milk administration prevented streptozotocin (45 mg/kg, administered intraperitoneally)-induced type 2 diabetes mellitus and associated oxidative stress in experimental rat models. The protective effects were mediated through the enhancement of endogenous antioxidant enzyme activities, specifically GSH, CAT, and SOD, while concurrently reducing MDA concentrations, a biomarker of lipid peroxidation [135].

Heat stress exposure significantly compromised male reproductive function in murine models through multiple pathophysiological mechanisms, including increased testicular oxidative stress, altered gene expression profiles characterized by upregulation of tumor suppressor protein p53 and Nrf2, downregulation of B-cell lymphoma 2 (Bcl-2) and peroxisome proliferator-activated receptor-γ (PPAR-γ), induction of Leydig cell hyperplasia, and subsequent reductions in testosterone concentrations and sperm motility parameters [147]. Camel whey protein supplementation effectively restored testicular antioxidant homeostasis and normalized dysregulated gene expression patterns in heat-stressed experimental animals [147]. The therapeutic intervention successfully reversed Leydig cell dysfunction, resulting in restored testosterone biosynthesis and significantly improved sperm motility parameters. These findings demonstrate that camel whey protein confers protective effects against heat stress-induced testicular damage through molecular mechanisms involving Leydig cell functional restoration and antioxidant system rehabilitation [147]. These findings collectively demonstrate that camel milk and its derived bioactive compounds possess significant therapeutic potential through multiple mechanisms, including antioxidant activity, anti-inflammatory effects, and neuroprotective properties, warranting further clinical investigation for various pathological conditions. Table 3 provides a comprehensive summary of the documented biological activities associated with camel milk, along with corresponding literature references.

## 7. Health Benefits of Camel Milk

Camel milk has a distinctive composition featuring unique protein structures (including smaller, more stable nanobodies), higher levels of vitamin C and beneficial fatty acids, enhanced immunological components like lactoferrin and immunoglobulins, and notably lacks β-lactoglobulin, which makes it less allergenic than cow’s milk. This unique profile, combined with various bioactive peptides and superior mineral content, makes it particularly valuable for research into therapeutic applications. Compared with bovine milk, it contains higher levels of antimicrobial factors, immunoglobulins, and bioactive compounds [161]. Its nutritional profile includes lower fat content with more polyunsaturated fatty acids, comparable protein levels with distinctive composition, higher vitamin C concentration, significant minerals, and lower lactose content beneficial for those with lactose intolerance [162]. Published research studies reveal that camel milk protein hydrolysates demonstrate remarkable bio-functional properties. These bioactive compounds exhibit significant antioxidant capabilities while simultaneously displaying antidiabetic effects. The hydrolysates further demonstrate anticancer potential alongside antiallergic responses and hepatoprotective functions. In parallel, these compounds show pronounced anti-inflammatory actions and antimicrobial activity. Their ACE inhibitory properties work in conjunction with antiradical scavenging abilities and anti-autism therapeutic activities. This comprehensive range of biological functions underscores the therapeutic potential of camel milk protein hydrolysates. Such properties establish them as valuable functional food ingredients and nutraceutical compounds across diverse health applications [18,163,164,165,166,167]. The anti-inflammatory and antioxidant properties of camel milk stem from its ability to inhibit pro-inflammatory cytokines, modulate NF-κB pathways, reduce inflammatory mediators, and neutralize reactive oxygen species through its rich content of antioxidant enzymes [7,131]. These properties make it potentially beneficial for inflammatory disorders.

Studies have demonstrated camel milk’s antidiabetic effects, including improved insulin sensitivity, enhanced β-cell function, and reduced blood glucose levels [168,169]. The presence of insulin-like proteins that survive gastric digestion may contribute to these effects. Preliminary clinical research from small-scale studies suggests regular consumption (500 mL/day for 2–3 months) may reduce insulin requirements in type 1 diabetes patients by 30–35%. However, these findings require validation through larger, controlled trials before becoming standard clinical recommendations [170]. Camel milk shows anticancer potential through antiproliferative effects against various cancer cell lines, induction of apoptosis, cell cycle arrest, and inhibition of angiogenesis [9]. Its antimicrobial properties are effective against various bacteria, fungi, and some viruses due to protective proteins like lysozyme and lactoferrin [171].

Research indicates camel milk offers renoprotective effects by attenuating drug-induced nephrotoxicity and reducing kidney injury markers [25]. Its immunomodulatory properties enhance natural killer cell activity, balance T-helper responses, and regulate inflammatory cytokines, potentially benefiting autoimmune conditions [40]. Furthermore, camel milk has also been reported to alleviate several health issues, including inflammatory bowel disease [145], rheumatoid arthritis [3], steatohepatitis [172], and wound healing [173]. Significantly, the pronounced anti-inflammatory properties of camel milk, characterized by reduced levels of pro-inflammatory cytokines and enhanced anti-inflammatory mediator expression, have been demonstrated to provide substantial nephroprotective effects across multiple experimental rodent models of kidney injury [27,144]. These beneficial outcomes have been particularly well-documented in animal studies examining diabetes mellitus-induced diabetic nephropathy and chemotherapy-induced acute kidney injury, where camel milk supplementation has shown measurable improvements in renal function parameters, histopathological changes, and biomarkers of oxidative stress and inflammation [27,144].

**Figure 4:**
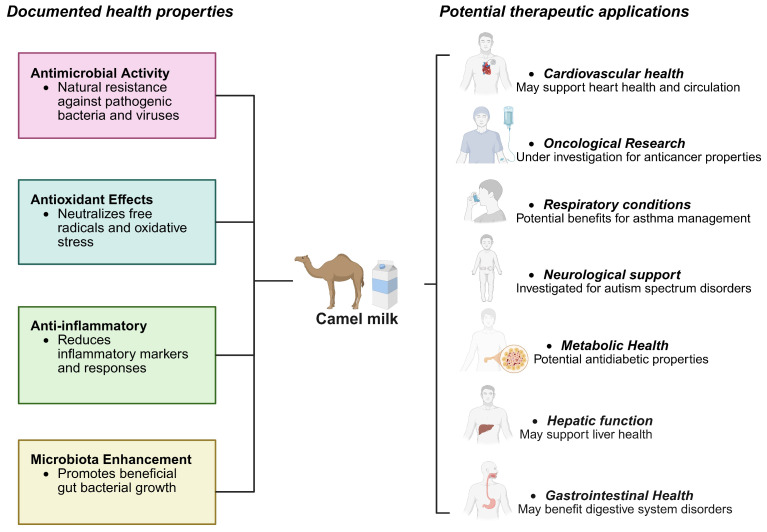
Camel milk—health properties and potential therapeutic applications. This figure presents a clear distinction between established health properties of camel milk (**left panel**) and potential therapeutic applications under investigation (**right panel**). The central positioning emphasizes camel milk as the source of both documented properties and research interests while avoiding implications of direct treatment recommendations. Important Note: The therapeutic applications shown represent areas of ongoing research and should not be interpreted as medical recommendations. Further clinical studies are needed to establish efficacy and safety profiles.

Camel milk benefits gastrointestinal health by modulating gut microbiota, enhancing intestinal barrier function, and reducing inflammation. It also demonstrates hepatoprotective potential by reducing liver enzymes and oxidative stress markers in liver injury models. Despite promising evidence, challenges remain, including standardizing milk composition, conducting large-scale clinical trials, determining optimal dosages, and understanding specific mechanisms of action. Future investigations should prioritize the identification and isolation of bioactive compounds, with particular emphasis on characterizing peptides such as lactoferrin-derived fragments. Critical research objectives include elucidating the pharmacokinetic profiles and bioavailability of these compounds through rigorously designed controlled clinical trials. Essential research priorities include implementing randomized, double-blind, placebo-controlled human studies to establish therapeutic efficacy. Additionally, researchers should focus on developing evidence-based functional food formulations and systematically evaluating potential synergistic interactions with established conventional therapies. Camel milk represents a promising functional food with multiple health benefits supported by emerging scientific evidence, offering therapeutic potential for various health conditions through its anti-inflammatory, antioxidant, antidiabetic, antimicrobial, and immunomodulatory properties. The health benefits of camel milk have been summarized in Figure 4.

## 8. Conclusions

This comprehensive review demonstrates substantial evidence for the exceptional antioxidant and anti-inflammatory properties of camel and donkey milk, establishing their potential as valuable functional foods. Their unique nutritional profiles, characterized by elevated bioactive proteins, essential fatty acids, and minerals, contribute to superior therapeutic properties compared with conventional bovine milk. The health benefits are mediated through key pathways, including NF-κB inhibition, Nrf2-ARE activation, and favorable cytokine modulation. Donkey milk exhibits therapeutic potential for immunological disorders attributed to its elevated lysozyme concentrations. Similarly, camel milk has demonstrated clinical efficacy in diabetes management, with studies indicating 30–35% reductions in insulin requirements among patients. Nevertheless, comprehensive validation through large-scale population studies and randomized controlled trials remains essential to establish definitive therapeutic benefits and safety profiles prior to integration into standard clinical practice guidelines. Although these findings are promising, research limitations include compositional variability due to animal breed, diet, and geographical factors, which necessitates the development of standardization protocols. The limited number of large-scale clinical trials highlights the need for comprehensive human studies to validate therapeutic efficacy and establish optimal dosing regimens. Future research should prioritize randomized controlled trials and the characterization of specific bioactive compounds. Camel and donkey milk represent validated examples of traditional knowledge supported by modern scientific investigation. Their demonstrated bioactive profiles position them as valuable additions to functional foods, offering natural interventions for chronic disease management as healthcare increasingly embraces preventive and integrative approaches. The evidence strongly supports their therapeutic potential, warranting continued research for their integration into evidence-based healthcare strategies.

## Figures and Tables

**Figure 3:**
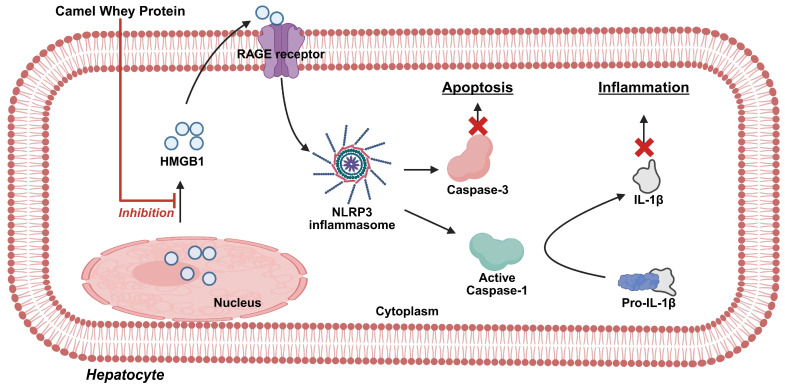
Molecular mechanism of prevention of hepatic inflammation and injury by camel milk. This figure illustrates how camel whey protein protects hepatocytes by blocking inflammation and apoptosis pathways. The diagram shows a liver cell where HMGB1 molecules normally bind to RAGE receptors, activating the NLRP3 inflammasome complex. This activation triggers two harmful responses: Caspase-1 converts Pro-IL-1β to mature IL-1β (promoting inflammation), and Caspase-3 activation leads to cell death. However, camel whey protein intervenes by inhibiting HMGB1 activity, as shown by the red inhibition arrow and X marks throughout the pathway. This blockade prevents both inflammatory cytokine production and apoptosis, demonstrating camel whey protein’s potential as a liver-protective therapeutic agent that simultaneously targets multiple pathological processes. The figure is adopted based on findings of Du et al. [157,158,159].

**Table 2:** Biological activities of donkey milk.

Donkey Milk/Derived Product	Biological Activities	Experimental Model	Reference
Intragastric administration of 10 mL/kg of body weight of donkey milk/28 days	✧ Enhanced immunity markers (IL-6 and TNF-α) in liver of mouse	Mouse model	[97]
Pretreatment of donkey milk for 10 days prior to ethanol induction	✧ Prevent ethanol-induced gastric ulcer ✧ Reduced MDA level and GSH expression in gastric tissue	Mouse model	[111]
*Lactobacillus* and *Enterococcus* (isolated from donkey milk)-derived exopolysaccharides	✧ Antimicrobial and immunoregulatory properties against *Salmonella typhimurium*	Cell culture model	[121]
*Lacticaseibacillus paracasei* (isolated from donkey milk)	✧ Showed a strong antimicrobial response against *staphylococcus aureus*	Cell culture model	[122]
Donkey milk-derived exosomes	✧ The milk-derived exosomes proteins associated with immunity (enhanced expression of ACTB, C3, ACTR2, ACTR3B, ARPC1B) and antioxidants activities 9 (increased GPx3 level)	Donkey	[123]
Donkey milk	✧ Donkey milk supplementation suppressed NF-κB signaling pathway by reducing levels of NF-κBp65 and p-NF-κB p65 proteins. ✧ Elevated level of miR-223-3p, thereby inhibiting the expression of IKKα and NF-κB proteins. ✧ The mRNA level of MCP-1 was reduced significantly ✧ mRNA expression levels of IL6, TNF-α, and IL-1β were also decreased ✧ Decreased expression of TGF-β1 and alleviate renal fibrosis	Sprague–Dawley rats	[124]
Donkey milk (2.0 g/kg/day)	✧ Improved clinical condition and preserved colonic cytoarchitecture in 2,4-dinitrobenzene sulfonic acid (DNBS)-induced colitis mice ✧ Increased intestinal barrier markers (Villin, trefoil factor 3, and occludin type 1) ✧ Suppressed pro-inflammatory markers (TNF-α, IL-1β, IL-17, iNOS, COX-2) and pathways (NF-ΚB p65, MAPK-1) while up-regulating anti-inflammatory markers (IL-10, SOCS-1) ✧ Demonstrated antioxidant activity and maintained intestinal microbiota homeostasis	Mouse model	[125]
Donkey milk whey proteins	✧ Donkey whey protein treatment showed a significant effect against dextran sulfate sodium-induced ulcerative colitis, comparing them to bovine whey proteins using proteomics and mouse models. ✧ Demonstrated superior anti-inflammatory effects by increasing regulatory T cell accumulation and CD205+ macrophage abundance compared with bovine proteins and control groups. ✧ The treatment significantly reduced pro-inflammatory protein expressions (S100A8, TRAF6, NF-κB) and inflammatory secretion more effectively than bovine whey proteins. ✧ These findings establish donkey whey proteins as a promising functional food ingredient for colitis treatment and provide groundwork for future therapeutic research.	Mouse and cell culture models	[126]
Donkey milk-derived lactic acid bacteria	✧ *Lactobacillus plantarum* M2 and KO9, isolated from donkey and mare milk, demonstrated excellent probiotic characteristics, including resistance to gastrointestinal conditions, antimicrobial activity against pathogens, and strong aggregation abilities. ✧ The extracellular metabolites (molecular weight <2000 Da) from both strains significantly suppressed TNF-α production by up to 67% in LPS-stimulated human PBMCs, demonstrating potent anti-inflammatory effects. ✧ The metabolites exhibited no cytotoxic or genotoxic effects on PBMCs, confirming their safety profile alongside their therapeutic anti-inflammatory potential.	Cell culture model	[127]
Donkey milk	✧ Allergic asthma involves airway hyperresponsiveness, remodeling, mucus hypersecretion, and Th2 inflammatory responses, while donkey milk possesses anti-inflammatory properties similar to human milk composition. ✧ Oral administration of donkey milk powder at doses of 0.4, 2, and 10 g/kg effectively ameliorated airway hyperresponsiveness and delayed asthma manifestation in ovalbumin-challenged mice. ✧ Donkey milk powder treatment reduced airway epithelial injury and fibrosis, decreased MUC5AC mucin accumulation and goblet cell hyperplasia, and downregulated MUC5AC expression in human airway epithelial cells. ✧ The intervention also reduced airway eosinophilia, decreased Th2 cytokines in bronchoalveolar lavage fluid, lowered serum immunoglobulin E levels, and inhibited NF-κB-P65 activity, demonstrating preventive effects against ovalbumin-induced asthma.	Mouse model	[128]

Note: NF-κB (nuclear factor kappa-B); IKKα (inhibitor of kappa-B kinase); ACTB (β-actin); C3 (complement component 3); ACTR2 (actin related protein 2); ACTR3B (actin related protein 3B); ARPC1B (actin related protein 2/3 complex subunit 1B); IL-6 (interleukin-6); TNF-α (tumor necrosis factor-alpha); Nrf2 (nuclear factor erythroid 2-related factor 2); MCP-1 (monocyte chemoattractant protein-1); iNOS (inducible nitric oxide synthase); COX-2 (cyclooxygenase-2); MAPK-1 (mitogen-activated protein kinase 1); SOCS-1 (suppressor of cytokine signaling 1); S100A8 (S100 calcium-binding protein A8); TRAF6 (TNF receptor associated factor 6); MUC5AC (mucin 5AC).

**Table 3:** Biological activities of camel milk.

Camel Milk	Biological Activities	Experimental Model	Reference
Camel milk (10 mL/kg)	✧ Effectively mitigates cyclosporine A (CsA)-induced nephrotoxicity in rats by reducing key nephrotoxicity markers such as creatinine, BUN, and NGAL. ✧ Attenuates renal oxidative stress by enhancing antioxidant levels and modulating the Nrf2/HO-1 pathway. ✧ Reduces renal apoptosis and promotes tissue repair by regulating pro-apoptotic and anti-apoptotic markers. ✧ Activates the PI3K/AKT/eNOS/NO pathway, further supporting its nephroprotective potential.	Rats	[25]
Camel milk (10 mL/kg/day for 3 weeks by gavage)	✧ Prevented cyclosporine-induced renal inflammation and consequent damage. ✧ Decreased the expression of pro-inflammatory cytokines (MCP-1, TNF-α, IL-1β, and IL-18). ✧ Promoted the level of IL10. ✧ Inhibited the phosphorylation of MAPK and NF-κB signaling pathway activation. ✧ Camel milk inhibited cyclosporine-induced renal oxidative stress by lowering the MPO activity and augmenting the reduced/oxidized glutathione ratio and total antioxidant capacity.	Rats	[26]
Intragastric administration of camel milk	✧ Camel milk ameliorates alcohol-induced liver injury. ✧ Enhanced antioxidant response (decreased MDA level and improved SOD and CAT levels. ✧ Decreased level of TNF-a, IL-6, and IL-1β and enhanced the level of IL-10.	Mouse model	[142]
Camel milk	✧ Relieved the alcohol-induced liver injury. ✧ Enhanced antioxidant response and ameliorated oxidative stress. ✧ Prevent inflammatory changes and enhanced microbiota.	Rats	[143]
Camel milk (10 mL/kg b.i.d. by oral gavage)	✧ Prevented inflammatory bowel diseases (IBD) by decreasing inflammatory cytokines.	Rats	[145]
Camel milk-derived lactoferrin (250 mg/day)	✧ Camel milk lactoferrin supplementation demonstrated significant prevention of type 2 diabetes. ✧ Improved lipid profiles, accompanied by upregulated PPAR-γ and SIRT-1 expression. ✧ Decreased pro-inflammatory cytokines (IL-1β, IL-6, IL-18, TNF-α) and lipocalin-2 levels, correlating with reduced NF-κB signaling activation. ✧ Enhanced antioxidant capacity was confirmed through elevated SOD activity and Nrf2 expression.	Type 2 diabetic patient	[146]
Camel whey protein	✧ Ameliorated heat stress-induced testicular damage.	Mice	[147]
Camel whey protein	✧ Prevented acute heat stress-induced renal injury via activating PI3K/AKT/eNOS signaling pathway. ✧ Enhanced antioxidant response (increased level of SOD, GSH-PX, CAT, and T-AOC and decreased MDA level).	Rats	[148]
Camel milk	✧ Prevented cigarette smoke (CS)-induced chronic obstructive pulmonary disease, inflammation, and oxidative stress. ✧ Reduced level of TNF-*α* and malondialdehyde (MDA) level in serum and homogenized tissues of the heart, kidney, liver, and testicle. ✧ Enhanced levels of CAT and SOD and thiol levels were significantly decreased in CS-exposed rats.	Rats	[149]
Camel milk protein hydrolysates (500 mg/kg of BW) for 8 wks	✧ Camel milk protein hydrolysates controlled hyperglycemia and hyperlipidemia and prevented oxidative stress in streptozotocin (STZ)-induced diabetic rats. ✧ Improved CAT, SOD, and GPX as well as reduced MDA level.	Rats	[150]
Camel milk in combination with plant sterol	✧ Inhibited level of low-density lipoprotein (LDL) and oxidative stress (MDA decreased). ✧ Reduced chances of atherogenesis and cardiovascular diseases.	Rats	[151]
Camel milk (1 mL of milk/kg body weight).	✧ Prevent aflatoxin B1-induced hepatic injury in rats. ✧ Relieved inflammation and oxidative stress by suppressing tumor necrosis factor (TNF-α) and enhancing (NAD(P)H quinone oxidoreductase 1 (NQO1)) and base excision repair genes (APE1 and OGG1) expression in the liver tissue.	Rats	[152,153]
Camel milk (1 mL of camel milk/kg body weight orally)	✧ Improved testicular damage induced by aflatoxin B1 following the enhancement of tumor necrosis factor (TNF-α), luteinizing hormone receptor (LHR), and steroidogenic acute regulatory protein (StAR) in the rats’ testes.	Rats	[154]
Camel milk protein hydrolysate	✧ Prevent hepatic steatosis and hypertension in high-fructose-fed rats by reducing cholesterol and triglyceride levels in serum and liver. ✧ ALT and AST, angiotensin II, ACE, and endothelin-1 levels in serum. ✧ Camel milk protein hydrolysate inhibited hepatic fat deposition in the hepatocytes and decreased the chances of hepatocyte damage by reducing mRNA expression of AMPK, increasing PPARα in hepatic cells, and enhancing the level of fructokinase C, SREBP1, SREBP2, fatty acid synthase, and HMG-CoA-reductase.	Rats	[155]
Camel milk (50 mg/kg of body weight)	✧ Controlled streptozotocin (STZ)-induced type 1 diabetic albino rats. ✧ Ameliorated oxidative stress by enhancing the levels of catalase, GPx, and SOD in pancreas and normalized insulin levels.	Rats	[156]
Camel milk	✧ Alleviated HS-induced hepatocyte oxidative stress by decreasing ROS and NO and enhancing SOD, CAT, GSH-Px, and HO-1 activities. ✧ Decreased the phosphorylation of NF-κB p65, suppressed the expression of NLRP3 and caspase-1, and alleviated caspase-3-mediated apoptosis. ✧ By activating the Nrf2/HO-1 signaling pathway and inhibiting NF-κB/NLRP3 camel milk prevented the hepatocytes from being damaged.	Hepatocytes	[157]
Camel whey protein	✧ Heat stress (HS)-induced liver injury was prevented via inhibition of NLRP3 inflammasome via activation of HMGB1/RAGE signaling pathway. ✧ Camel milk whey protein suppresses activation of NLRP3 to restore HMGB1 nuclear localization. ✧ Reversed HS-induced abnormal expression of HMGB1, RAGE, NLRP3, IL-1β, ALT, Bcl-2, and Caspase-3, inhibited Caspase-1 activity, and alleviated apoptosis and subsequent liver injury (Figure 3).	Rats	[158]
Camel whey protein	✧ Camel whey protein prevents high-mobility group box 1 (HMGB1) protein release from liver cells during heat stress. ✧ Reduces inflammation by lowering NLRP3 inflammasome, IL-1β, and TNF-α levels. ✧ Works through antioxidant activity via the Nrf2/HO-1 signaling pathway. ✧ Ameliorated heat stress-induced liver injury.	Rats	[159]
Camel milk-derived lactic acid bacteria	✧ Significantly reduced liver damage and decreased inflammatory markers (AST, ALT, TNF-α, IL-6) compared with untreated controls. ✧ *Lactobacillus paracasei* subsp. *paracasei* WXD5 showed the strongest protective effects, demonstrating its potential as a therapeutic intervention for inflammation-based liver diseases.	Rats	[160]

## Data Availability

The data that support the findings of this study are available in manuscript.

## References

[1] Xie A., Shen X., Hong R., Xie Y., Zhang Y., Chen J., Li Z., Li M., Yue X., Quek S.Y. (2025). Unlocking the potential of donkey milk: Nutritional composition, bioactive properties and future prospects. Food Res. Int..

[2] Amr M., Farid A. (2024). Impact of cow, buffalo, goat or camel milk consumption on oxidative stress, inflammation and immune response post weaning time. Sci. Rep..

[3] Cimmino F., Catapano A., Villano I., Di Maio G., Petrella L., Traina G., Pizzella A., Tudisco R., Cavaliere G. (2023). Invited review: Human, cow, and donkey milk comparison: Focus on metabolic effects. J. Dairy Sci..

[4] Salvo E.D., Conte F., Casciaro M., Gangemi S., Cicero N. (2023). Bioactive natural products in donkey and camel milk: A perspective review. Nat. Prod. Res..

[5] Derdak R., Sakoui S., Pop O.L., Muresan C.I., Vodnar D.C., Addoum B., Vulturar R., Chis A., Suharoschi R., Soukri A. (2020). Insights on health and food applications of *Equus asinus* (donkey) milk bioactive proteins and peptides—An overview. Foods.

[6] Khan M.Z., Chen W., Li M., Ren W., Huang B., Kou X., Ullah Q., Wei L., Wang T., Khan A. (2024). Is there sufficient evidence to support the health benefits of including donkey milk in the diet?. Front. Nutr..

[7] Kocyigit E., Abdurakhmanov R., Kocyigit B.F. (2024). Potential role of camel, mare milk, and their products in inflammatory rheumatic diseases. Rheumatol. Int..

[8] Garhwal R., Sangwan K., Mehra R., Kumar N., Bhardwaj A., Pal Y., Buttar H.S., Kumar H. (2022). A systematic review of the bioactive components, nutritional qualities and potential therapeutic applications of donkey milk. J. Equine Vet. Sci..

[9] Khan M.Z., Xiao J., Ma Y., Ma J., Liu S., Khan A., Khan J.M., Cao Z. (2021). Research development on anti-microbial and antioxidant properties of camel milk and its role as an anti-cancer and anti-hepatitis agent. Antioxidants.

[10] Keitshweditse B., Tsvakirai C.Z., Mabuza M.L., Tshehla M. (2024). Towards the expansion of the functional dairy market: Determining donkey milk value propositions and identifying possible consumers. Future Foods..

[11] Oselu S., Ebere R., Arimi J.M. (2022). Camels, camel milk, and camel milk product situation in Kenya in relation to the world. Int. J. Food Sci..

[12] Singh M.P., Vashisht P., Singh L., Awasti N., Sharma S., Mohan C., Singh T.P., Sharma S., Shyam S., Charles A.P. (2024). Donkey milk as a non-bovine alternative: A review of its nutri-functional properties, applications, and challenges. J. Food Sci. Technol..

[13] Chen Y.Z., Li C., Gu J., Lv S.C., Song J.Y., Tang Z.B., Duan G.X., Qin L.Q., Zhao L., Xu J.Y. (2021). Anti-oxidative and immuno-protective effect of camel milk on radiation-induced intestinal injury in C57BL/6 J mice. Dose Response.

[14] Polidori P., Cammertoni N., Santini G., Klimanova Y., Zhang J.J., Vincenzetti S. (2021). Nutritional properties of camelids and equids fresh and fermented milk. Dairy.

[15] Dharmisthaben P., Basaiawmoit B., Sakure A., Das S., Maurya R., Bishnoi M., Kondepudi K.K., Hati S. (2021). Exploring potentials of antioxidative, anti-inflammatory activities and production of bioactive peptides in lactic fermented camel milk. Food Biosci..

[16] Arab H.H., Salama S.A., Abdelghany T.M., Omar H.A., Arafa E.S., Alrobaian M.M., Maghrabi I.A. (2017). Camel milk attenuates rheumatoid arthritis via inhibition of mitogen activated protein kinase pathway. Cell. Physiol. Biochem..

[17] Badawy A.A., El-Hofey S.M., Shaban A.M., Orif S.E., Uyanıkgil Y., El-Magd M.A. (2025). Camel milk extracellular vesicles/exosomes: A fascinating frontier in isolation and therapeutic potential. Food Funct..

[18] Arain M.A., Khaskheli G.B., Shah A.H., Marghazani I.B., Barham G.S., Shah Q.A., Khand F.M., Buzdar J.A., Soomro F., Fazlani S.A. (2023). Nutritional significance and promising therapeutic/medicinal application of camel milk as a functional food in human and animals: A comprehensive review. Anim. Biotechnol..

[19] Behrouz S., Saadat S., Memarzia A., Sarir H., Folkerts G., Boskabady M.H. (2022). The antioxidant, anti-inflammatory and immunomodulatory effects of camel milk. Front. Immunol..

[20] Li Y., Ma Q., Liu G., Wang C. (2022). Effects of donkey milk on oxidative stress and inflammatory response. J. Food Biochem..

[21] Al-Ayadhi L., Alhowikan A.M., Bhat R.S., El-Ansary A. (2022). Comparative study on the ameliorating effects of camel milk as a dairy product on inflammatory response in autism spectrum disorders. Neurochem. J..

[22] Akan E. (2021). An evaluation of the in vitro antioxidant and antidiabetic potentials of camel and donkey milk peptides released from casein and whey proteins. J. Food Sci. Technol..

[23] Demir R., Sarıtaş S., Bechelany M., Karav S. (2025). Lactoferrin: Properties and potential uses in the food industry. Int. J. Mol. Sci..

[24] Musa K.H., Hamad E.M., Elnour A.A. (2025). Camel milk and oxidative stress: Therapeutic potential against metabolic syndrome diseases. J. Agric. Food Res..

[25] Arab H.H., Eid A.H., Gad A.M., Yahia R., Mahmoud A.M., Kabel A.M. (2021). Inhibition of oxidative stress and apoptosis by camel milk mitigates cyclosporine-induced nephrotoxicity: Targeting Nrf2/HO-1 and AKT/eNOS/NO pathways. Food Sci. Nutr..

[26] Arab H.H., Ashour A.M., Alqarni A.M., Arafa E.S.A., Kabel A.M. (2021). Camel milk mitigates cyclosporine-induced renal damage in rats: Targeting p38/ERK/JNK MAPKs, NF-κB, and matrix metalloproteinases. Biology.

[27] Arab H.H., Salama S.A., Maghrabi I.A. (2018). Camel milk ameliorates 5-fluorouracil-induced renal injury in rats: Targeting MAPKs, NF-κB and PI3K/Akt/eNOS pathways. Cell. Physiol. Biochem..

[28] Heurtaux T., Bouvier D.S., Benani A., Helgueta Romero S., Frauenknecht K.B., Mittelbronn M., Sinkkonen L. (2022). Normal and pathological NRF2 signalling in the central nervous system. Antioxidants.

[29] Thanas C., Ziros P.G., Chartoumpekis D.V., Renaud C.O., Sykiotis G.P. (2020). The Keap1/Nrf2 signaling pathway in the thyroid—2020 update. Antioxidants.

[30] Li R., Jia Z., Zhu H. (2019). Regulation of Nrf2 signaling. React. Oxyg. Species.

[31] Khan M.Z., Li L., Zhan Y., Binjiang H., Liu X., Kou X., Khan A., Qadeer A., Ullah Q., Alzahrani K.J. (2025). Targeting Nrf2/KEAP1 signaling pathway using bioactive compounds to combat mastitis. Front. Immunol..

[32] Yuan C., Li H., Zhang M., Wang Z., Dong J., Cui L., Guo L., Liu K., Li J., Wang H. (2025). Selenium yeast alleviates Escherichia coli-induced endometritis in goats under high cortisol background. Animals.

[33] Song P., Liu C., Sun M., Liu J., Lin P., Chen H., Zhou D., Tang K., Wang A., Jin Y. (2023). Transcription factor Nrf2 modulates lipopolysaccharide-induced injury in bovine endometrial epithelial cells. Int. J. Mol. Sci..

[34] Khan M.Z., Chen W., Liu X., Kou X., Khan A., Khan R.U., Zahoor M., Wang C. (2024). An overview of bioactive compounds’ role in modulating the Nrf2/Keap1/NF-κB pathway to alleviate lipopolysaccharide-induced endometritis. Int. J. Mol. Sci..

[35] Rilwan H.B., Adebisi S.S., Timbuak J.A., Oladele S.B., Muhammad A., Sadeeq A.A., Makena W. (2022). Camel milk ameliorates diabetes in pigs by preventing oxidative stress, inflammation and enhancing beta cell function. J. Diabetes Metab. Disord..

[36] Albertos I., López M., Jiménez J.M., Cao M.J., Corell A., Castro-Alija M.J. (2022). Characterisation of Zamorano-Leonese donkey milk as an alternative sustainably produced protein food. Front. Nutr..

[37] Kaskous S., Pfaffl M.W. (2022). Milk properties and morphological characteristics of the donkey mammary gland for development of an adopted milking machine—A review. Dairy.

[38] Basdeki A.M., Fatouros D.G., Biliaderis C.G., Moschakis T. (2021). Physicochemical properties of human breast milk during the second year of lactation. Curr. Res. Food Sci..

[39] Arain M.A., Salman H.M., Ali M., Khaskheli G.B., Barham G.S., Marghazani I.B., Ahmed S. (2024). A review on camel milk composition, techno-functional properties and processing constraints. Food Sci. Anim. Resour..

[40] Swelum A.A., El-Saadony M.T., Abdo M., Ombarak R.A., Hussein E.O.S., Suliman G., Alhimaidi A.R., Ammari A.A., Ba-Awadh H., Taha A.E. (2021). Nutritional, antimicrobial and medicinal properties of camel’s milk: A review. Saudi J. Biol. Sci..

[41] Khaliq A., Mishra A.K., Niroula A., Baba W.N., Shaukat M.N., Rabbani A. (2024). An updated comprehensive review of camel milk: Composition, therapeutic properties, and industrial applications. Food Biosci..

[42] Meena S., Meena G.S., Gautam P.B., Rai D.C., Kumari S. (2024). A comprehensive review on donkey milk and its products: Composition, functionality and processing aspects. Food Chem. Adv..

[43] Zou Z., Bauland J., Hewavitharana A.K., Al-Shehri S.S., Duley J.A., Cowley D.M., Koorts P., Shaw P.N., Bansal N. (2021). A sensitive, high-throughput fluorescent method for the determination of lactoperoxidase activities in milk and comparison in human, bovine, goat and camel milk. Food Chem..

[44] Verardo V., Gómez-Caravaca A.M., Arráez-Román D., Hettinga K. (2017). Recent advances in phospholipids from colostrum, milk and dairy by-products. Int. J. Mol. Sci..

[45] Khan I.T., Nadeem M., Imran M., Ullah R., Ajmal M., Jaspal M.H. (2019). Antioxidant properties of milk and dairy products: A comprehensive review of the current knowledge. Lipids Health Dis..

[46] Moatsou G., Sakkas L. (2019). Sheep milk components: Focus on nutritional advantages and biofunctional potential. Small Rumin. Res..

[47] Siregar K., Purwati E., Kurnia Y., Melia S. (2021). Chemical properties of buffalo milk from Sianok Village, Agam District, West Sumatera, Indonesia. IOP Conference Series: Earth and Environmental Science.

[48] Becskei Z., Savić M., Ćirković D., Rašeta M., Puvača N., Pajić M., Đorđević S., Paskaš S. (2020). Assessment of water buffalo milk and traditional milk products in a sustainable production system. Sustainability.

[49] İpek S.L. (2024). Bacterial diversity and lysozyme activity of raw buffalo milk: A case study on milk collection tanks from selected farms. CyTA J. Food.

[50] Parmar A., Sharma V., Arora S., Raju Panjagari N. (2022). Activation of lactoperoxidase system in buffalo milk using dual enzyme (lactase & glucose oxidase) and its effect on milk constituents. Int. J. Dairy Technol..

[51] Vargas-Ramella M., Pateiro M., Maggiolino A., Faccia M., Franco D., De Palo P., Lorenzo J.M. (2021). Buffalo milk as a source of probiotic functional products. Microorganisms.

[52] Kazimierska K., Kalinowska-Lis U. (2021). Milk proteins—Their biological activities and use in cosmetics and dermatology. Molecules.

[53] Navarro F., Galan-Malo P., Pérez M.D., Abecia J.A., Mata L., Calvo M., Sánchez L. (2018). Lactoferrin and IgG levels in ovine milk throughout lactation: Correlation with milk quality parameters. Small Rumin. Res..

[54] Moatsou G., Moschopoulou E., Zoidou E., Kamvysi A., Liaskou D., Tsigkou V., Sakkas L. (2021). Changes in native whey protein content, gel formation, and endogenous enzyme activities induced by flow-through heat treatments of goat and sheep milk. Dairy.

[55] Fitriani N., Astuti P., Mona Airin C., Sarmin, Adianto N. (2022). Comparison of albumin/globulin (A/G) ratio between pregnant and lactation of thin-tail sheep. BIO Web Conf..

[56] Ge X., Zhang J., Hu J., Liu D., Gao Y., Peng X., Wang S., Wang J., Li W., Zhou P. (2024). A comparative study of structural and digestive properties of bovine, goat and human α-lactalbumin with different calcium binding levels. Food Biosci..

[57] Landi N., Ragucci S., Di Maro A. (2021). Amino acid composition of milk from cow, sheep and goat raised in Ailano and Valle Agricola, two localities of ‘Alto Casertano’ (Campania Region). Foods.

[58] Wang X., Zhu H., Zhang W., Zhang Y., Zhao P., Zhang S., Pang X., Vervoort J., Lu J., Lv J. (2022). Triglyceride and fatty acid composition of ruminants milk, human milk, and infant formulae. J. Food Compos. Anal..

[59] Günay E., Güneşer O., Karagül Yüceer Y. (2021). A comparative study of amino acid, mineral and vitamin profiles of milk from Turkish Saanen, Hair and Maltese goat breeds throughout lactation. Int. J. Dairy Technol..

[60] Verma A.K., Singh T.P., Rajkumar V., Chatli M.K., Kushwah T., Nanda P.K., Curros B., Lorenzo J.M., Das A.K. (2025). Goat milk: A versatile dairy alternative with unique health benefits and functional properties. Food Rev. Int..

[61] Fantuz F., Ferraro S., Todini L., Cimarelli L., Fatica A., Marcantoni F., Salimei E. (2020). Distribution of calcium, phosphorus, sulfur, magnesium, potassium, and sodium in major fractions of donkey milk. J. Dairy Sci..

[62] Liu C., Liu L.X., Yang J., Liu Y.G. (2023). Exploration and analysis of the composition and mechanism of efficacy of camel milk. Food Biosci..

[63] Konuspayeva G., Faye B., Bengoumi M. (2022). Mineral status in camel milk: A critical review. Anim. Front..

[64] Vincenzetti S., Cammertoni N., Rapaccetti R., Santini G., Klimanova Y., Zhang J.-J., Polidori P. (2022). Nutraceutical and Functional Properties of Camelids’ Milk. Beverages.

[65] Vincenzetti S., Santini G., Polzonetti V., Pucciarelli S., Klimanova Y., Polidori P. (2021). Vitamins in human and donkey milk: Functional and nutritional role. Nutrients.

[66] Martini M., Altomonte I., Licitra R., Salari F. (2018). Technological and seasonal variations of vitamin D and other nutritional components in donkey milk. J. Dairy Sci..

[67] Malacarne M., Criscione A., Franceschi P., Bordonaro S., Formaggioni P., Marletta D., Summer A. (2019). New insights into chemical and mineral composition of donkey milk throughout nine months of lactation. Animals.

[68] Bzikowska-Jura A., Wesołowska A., Sobieraj P., Michalska-Kacymirow M., Bulska E., Starcevic I. (2023). Maternal diet during breastfeeding in correlation to calcium and phosphorus concentrations in human milk. J. Hum. Nutr. Diet..

[69] Wijesinha-Bettoni R., Burlingame B., Muehlhoff E., Bennett A., McMahon D. (2013). Milk and dairy products composition. Milk and Dairy Products in Human Nutrition.

[70] Faye B., Konuspayeva G., Bengoumi M. (2019). Vitamins of camel milk: A comprehensive review. J. Camelid Sci..

[71] Li M., Zhu Q., Hong R., Feng D., Liu Y., Yue X. (2022). Comprehensive characterization of donkey milk serum proteins. J. Future Foods.

[72] Li M., Yu H., Chen J., Abdlla R., Liu A., Song W., Li Q. (2021). Novel insights into whey protein differences between donkey and bovine milk. Food Chem..

[73] Li M., Zheng K., Song W., Yu H., Zhang X., Yue X., Li Q. (2021). Quantitative analysis of differentially expressed milk fat globule membrane proteins between donkey and bovine colostrum based on high-performance liquid chromatography with tandem mass spectrometry proteomics. J. Dairy Sci..

[74] Martini M., Salari F., Licitra R., La Motta C., Altomonte I. (2019). Lysozyme activity in donkey milk. Int. Dairy J..

[75] Licitra R., Chessa S., Salari F., Gattolin S., Bulgari O., Altomonte I., Martini M. (2019). Milk protein polymorphism in Amiata donkey. Livest. Sci..

[76] Addo C.N.A., Ferragut V. (2015). Evaluating the ultra-high pressure homogenization (UHPH) and pasteurization effects on the quality and shelf life of donkey milk. Int. J. Food Stud..

[77] Martini M., Altomonte I., Licitra R., Salari F. (2018). Nutritional and nutraceutical quality of donkey milk. J. Equine Vet. Sci..

[78] Lönnerdal B., Erdmann P., Thakkar S.K., Sauser J., Destaillats F. (2017). Longitudinal evolution of true protein, amino acids and bioactive proteins in breast milk: A developmental perspective. J. Nutr. Biochem..

[79] Mao X., Gu J., Sun Y., Xu S., Zhang X., Yang H., Ren F. (2009). Anti-proliferative and anti-tumour effect of active components in donkey milk on A549 human lung cancer cells. Int. Dairy J..

[80] Licitra R., Li J., Liang X., Altomonte I., Salari F., Yan J., Martini M. (2019). Profile and content of sialylated oligosaccharides in donkey milk at early lactation. LWT.

[81] Garhwal R., Bhardwaj A., Kumar H., Sangwan K., Kumari A., Bhavya, Singal M., Nayan V., Pal Y., Bhattacharya T.K. (2025). Biochemical, dielectric and surface characteristics of freeze-dried donkey milk powder. Food Agric. Immunol..

[82] Martini M., Altomonte I., Tricò D., Lapenta R., Salari F. (2021). Current knowledge on functionality and potential therapeutic uses of donkey milk. Animals.

[83] Arain M.A., Khaskheli G.B., Barham G.S., Marghazani I.B. (2024). Lactoferrin’s role in modulating NF-κB pathway to alleviate diabetes-associated inflammation: A novel in-silico study. Heliyon.

[84] Yami H.A., Tahmoorespur M., Javadmanesh A., Tazarghi A., Sekhavati M.H. (2023). The immunomodulatory effects of lactoferrin and its derived peptides on NF-κB signaling pathway: A systematic review and meta-analysis. Immun. Inflamm. Dis..

[85] Bodur M., Yilmaz B., Agagunduz D., Ozogul Y. (2025). Immunomodulatory effects of omega-3 fatty acids: Mechanistic insights and health implications. Mol. Nutr. Food Res..

[86] Durkin L.A., Childs C.E., Calder P.C. (2021). Omega-3 polyunsaturated fatty acids and the intestinal epithelium—A review. Foods.

[87] Jiang L., Lv J.Y., Liu J.W., Hao X.H., Ren F.Z., Guo H.Y. (2018). Donkey milk lysozyme ameliorates dextran sulfate sodium-induced colitis by improving intestinal barrier function and gut microbiota composition. J. Funct. Foods.

[88] Amati L., Marzulli G., Martulli M., Tafaro A., Jirillo F., Pugliese V., Martemucci G., D’Alessandro A.G., Jirillo E. (2010). Donkey and goat milk intake and modulation of the human aged immune response. Curr. Pharm. Des..

[89] Li M., Dong Y., Li W., Shen X., Abdlla R., Chen J., Cao X., Yue X. (2022). Characterization and comparison of whey proteomes from bovine and donkey colostrum and mature milk. LWT.

[90] Gubić J., Tasić T., Tomić J., Torbica A. (2014). Determination of whey proteins profile in Balkan donkey’s milk during lactation period. J. Hyg. Eng. Des..

[91] Saric L.C., Saric B.M., Mandic A.I., Torbica A.M., Tomic J.M., Cvetkovic D.D., Okanović Đ.G. (2012). Antibacterial properties of domestic Balkan donkeys’ milk. Int. Dairy J..

[92] Brumini D., Criscione A., Bordonaro S., Vegarud G.E., Marletta D. (2016). Whey proteins and their antimicrobial properties in donkey milk: A brief review. Dairy Sci. Technol..

[93] Tidona F., Criscione A., Devold T.G., Bordonaro S., Marletta D., Vegarud G.E. (2014). Protein composition and micelle size of donkey milk with different protein patterns: Effects on digestibility. Int. Dairy J..

[94] Yvon S., Olier M., Leveque M., Jard G., Tormo H., Haimoud-Lekhal D.A., Peter M., Eutamène H. (2018). Donkey milk consumption exerts anti-inflammatory properties by normalizing antimicrobial peptides levels in Paneth’s cells in a model of ileitis in mice. Eur. J. Nutr..

[95] Li Y., Fan Y., Shaikh A.S., Wang Z., Wang D., Tan H. (2020). Dezhou donkey (*Equus asinus*) milk a potential treatment strategy for type 2 diabetes. J. Ethnopharmacol..

[96] Tafaro A., Magrone T., Jirillo F., Martemucci G., D’Alessandro A., Amati L. (2007). Immunological properties of donkeys milk: Its potential use in the prevention of atherosclerosis. Curr. Pharm. Des..

[97] Trinchese G., Cavaliere G., De Filippo C., Aceto S., Prisco M., Chun J.T., Penna E., Negri R., Muredda L., Demurtas A. (2018). Human milk and donkey milk, compared to cow milk, reduce inflammatory mediators and modulate glucose and lipid metabolism, acting on mitochondrial function and oleylethanolamide levels in rat skeletal muscle. Front. Physiol..

[98] Ning J., Chen J., Zhu Q., Luo X., Yue X. (2025). Peptidomics profiling reveals the functions of endogenous peptides and parent proteins in donkey colostrum and mature milk. J. Food Compos. Anal..

[99] Wang J., Ren W., Sun Z., Liu S., Han Z., Wang Y., Zeng Y., Meng J., Yao X. (2025). Impact of intragastric administration of donkey milk on mouse immunity utilizing gut microbiomics and plasma metabolomics. Front. Vet. Sci..

[100] Taghiloo S., Allahmoradi E., Sadeghian-Kiadehi S.F., Omrani-Nava V., Nazar E., Ebrahimzadeh M.A. (2021). Up-regulation of human immune system function by donkey’s milk. Braz. J. Pharm. Sci..

[101] Jirillo F., Magrone T. (2014). Anti-inflammatory and anti-allergic properties of donkey’s and goat’s milk. Endocr. Metab. Immune Disord. Drug Targets.

[102] Jirillo F., Jirillo E., Magrone T. (2010). Donkey’s and goat’s milk consumption and benefits to human health with special reference to the inflammatory status. Curr. Pharm. Des..

[103] Yvon S., Schwebel L., Belahcen L., Tormo H., Peter M., Haimoud-Lekhal D.A., Eutamene H., Jard G. (2019). Effects of thermized donkey milk with lysozyme activity on altered gut barrier in mice exposed to water-avoidance stress. J. Dairy Sci..

[104] Baloš M.Ž., Pelić D.L., Jakšić S., Lazić S. (2023). Donkey milk: An overview of its chemical composition and main nutritional properties or human health benefit properties. J. Equine Vet. Sci..

[105] Aspri M., Leni G., Galaverna G., Papademas P. (2018). Bioactive properties of fermented donkey milk, before and after in vitro simulated gastrointestinal digestion. Food Chem..

[106] Trinchese G., Cavaliere G., Canani R.B., Matamoros S., Bergamo P., De Filippo C., Aceto S., Gaita M., Cerino P., Negri R. (2015). Human, donkey and cow milk differently affects energy efficiency and inflammatory state by modulating mitochondrial function and gut microbiota. J. Nutr. Biochem..

[107] Piovesana S., Capriotti A.L., Cavaliere C., La Barbera G., Samperi R., Chiozzi R.Z., Laganà A. (2015). Peptidome characterization and bioactivity analysis of donkey milk. J. Proteom..

[108] Cunsolo V., Saletti R., Muccilli V., Gallina S., Di Francesco A., Foti S. (2017). Proteins and bioactive peptides from donkey milk: The molecular basis for its reduced allergenic properties. Food Res. Int..

[109] Tidona F., Sekse C., Criscione A., Jacobsen M., Bordonaro S., Marletta D., Vegarud G.E. (2011). Antimicrobial effect of donkeys’ milk digested in vitro with human gastrointestinal enzymes. Int. Dairy J..

[110] Zenezini Chiozzi R., Capriotti A.L., Cavaliere C., La Barbera G., Piovesana S., Samperi R., Laganà A. (2016). Purification and identification of endogenous antioxidant and ACE-inhibitory peptides from donkey milk by multidimensional liquid chromatography and nanoHPLC-high resolution mass spectrometry. Anal. Bioanal. Chem..

[111] Sami M., Azizi S., Kheirandish R., Ebrahimnejad H., Alizadeh S. (2025). Protective effects of donkey milk on ethanol-induced gastric ulcer in rat. Vet. Med. Sci..

[112] Lionetti L., Cavaliere G., Bergamo P., Trinchese G., De Filippo C., Gifuni G., Gaita M., Pignalosa A., Donizzetti I., Putti R. (2012). Diet supplementation with donkey milk upregulates liver mitochondrial uncoupling, reduces energy efficiency and improves antioxidant and antiinflammatory defences in rats. Mol. Nutr. Food Res..

[113] Harvey C.J., Thimmulappa R.K., Singh A., Blake D.J., Ling G., Wakabayashi N., Fujii J., Myers A., Biswal S. (2009). Nrf2-regulated glutathione recycling independent of biosynthesis is critical for cell survival during oxidative stress. Free Radic. Biol. Med..

[114] Huang F., Ma Z., Du X., Wang C., Liu G., Zhou M. (2025). Methionine alters the fecal microbiota and enhances the antioxidant capacity of lactating donkeys. Animals.

[115] Li M., Sun L., Du X., Zhao Y., Ren W., Man L., Zhu M., Liu G., Khan M.Z., Wang C. (2024). Characterization and discrimination of donkey milk lipids and volatiles across lactation stages. Food Chem. X.

[116] Li S.Y., Tong M.M., Li L., Hui F., Meng F.Z., Zhao Y.L., Guo Y.M., Guo X.Y., Shi B.L., Yan S.M. (2024). Rectal microbiomes and serum metabolomics reveal the improved effect of Artemisia ordosica crude polysaccharides on the lactation performance, antioxidant status, and immune responses of lactating donkeys. J. Dairy Sci..

[117] Tong M., Li S., Hui F., Meng F., Li L., Shi B., Zhao Y., Guo X., Guo Y., Yan S. (2024). Effects of dietary selenium yeast supplementation on lactation performance, antioxidant status, and immune responses in lactating donkeys. Antioxidants.

[118] Zhou M., Huang F., Du X., Liu G., Wang C. (2024). Fermented *Codonopsis pilosula* residue improved milk performance of lactating donkeys by enhancing antioxidant capacity and regulating metabolism. Front. Vet. Sci..

[119] Garhwal R., Bhardwaj A., Sangwan K., Mehra R., Pal Y., Nayan V., Iquebal M.A., Jaiswal S., Kumar H. (2023). Milk from Halari donkey breed: Nutritional analysis, vitamins, minerals, and amino acids profiling. Foods.

[120] Zhou M., Huang F., Du X., Wang C., Liu G. (2023). Microbial quality of donkey milk during lactation stages. Foods.

[121] Israyelyan A., Balabekyan T., Aleksanyan L., Sahakyan I., Gasparyan A., Tkhruni F. (2025). Investigation of new exopolysaccharides produced by strains of donkey milk. Iran. J. Microbiol..

[122] Greifová G., Drobná E., Olejníková P., Greif G., Greifová M. (2025). Isolation, characterisation and technological properties of raw donkey’s milk isolate, *Lacticaseibacillus paracasei*, compared to raw goat’s and cow’s milk isolates. Czech J. Food Sci..

[123] Liu Y., Ma Q., Khan M.Z., Wang M., Xiang F., Zhang X., Kou X., Li S., Wang C., Li Y. (2025). Proteomic Profiling of Donkey Milk Exosomes Highlights Bioactive Proteins with Immune-Related Functions. Int. J. Mol. Sci..

[124] Huimin C., Peng W., Man C., Hong L., Yuxin Z., Zhiyu P., Jianjun Y. (2025). Donkey milk supplementation alleviates renal fibrosis of chronic kidney disease by enhancing anti-inflammatory ability. J. Dairy Sci..

[125] Araújo E.D., de Souza Araújo D.F., Messias T.B., da Silva V.C., Andrade A.W., de Araújo A.A., de Araújo Júnior R.F., de Aragão Tavares E., de Oliveira C.J., Leite E.L. (2024). Preventive effect on intestinal inflammation and modulation of the microbiota of ‘Nordestino’ donkey milk in experimental DNBS-induced colitis in mice. Int. Dairy J..

[126] Li M., Li Q., Abdlla R., Chen J., Yue X., Quek S.Y. (2023). Donkey whey proteins ameliorate dextran sulfate sodium-induced ulcerative colitis in mice by downregulating the S100A8-TRAF6-NF-κB axis-mediated inflammatory response. Food Sci. Hum. Wellness.

[127] Kostelac D., Gerić M., Gajski G., Markov K., Domijan A.M., Čanak I., Jakopović Ž., Svetec I.K., Žunar B., Frece J. (2021). Lactic acid bacteria isolated from equid milk and their extracellular metabolites show great probiotic properties and anti-inflammatory potential. Int. Dairy J..

[128] Lu Y., Zhou Y., Lin Y., Li W., Tian S., Hao X., Guo H. (2021). Preventive effects of donkey milk powder on the ovalbumin-induced asthmatic mice. J. Funct. Foods.

[129] Wang Y., Liang Z., Shen F., Zhou W., Manaer T., Jiaerken D., Nabi X. (2023). Exploring the immunomodulatory effects and mechanisms of Xinjiang fermented camel milk-derived bioactive peptides based on network pharmacology and molecular docking. Front. Pharmacol..

[130] Ayoub M.A., Palakkott A.R., Ashraf A., Iratni R. (2018). The molecular basis of the anti-diabetic properties of camel milk. Diabetes Res. Clin. Pract..

[131] Almasri R.S., Bedir A.S., Ranneh Y.K., El-Tarabily K.A., Al Raish S.M. (2024). Benefits of camel milk over cow and goat milk for infant and adult health in fighting chronic diseases: A review. Nutrients.

[132] Shori A.B. (2024). Comparative analysis of Lactobacillus starter cultures in fermented camel milk: Effects on viability, antioxidant properties, and sensory characteristics. Foods.

[133] Ubaid S., Pandey S., Akhtar M.S., Rumman M., Singh B., Mahdi A.A. (2022). SIRT1 mediates neuroprotective and neurorescue effects of camel α-lactalbumin and oleic acid complex on rotenone-induced Parkinson’s disease. ACS Chem. Neurosci..

[134] Arain M.A., Khaskheli G.B., Barham G.S., Shah Q.A., Nabi F., Almutairi M.H., Almutairi B.O., Marghazani I.B. (2025). Exploring the anti-diabetic properties of camel milk: Effects on blood glucose, antioxidant defense, and organ histo-morphological features in rabbits. J. Mol. Histol..

[135] Alharbi Y.M., Alkhidhr M., Barakat H. (2025). Antioxidative and ameliorative properties of probiotic-enriched fermented and unfermented turmeric-camel milk in streptozotocin-induced diabetes and oxidative stress in rats. Int. J. Health Sci..

[136] Shukla P., Sakure A., Maurya R., Bishnoi M., Kondepudi K.K., Das S., Liu Z., Padhi S., Rai A.K., Hati S. (2023). Antidiabetic, angiotensin-converting enzyme inhibitory and anti-inflammatory activities of fermented camel milk and characterisation of novel bioactive peptides from lactic-fermented camel milk with molecular interaction study. Int. J. Dairy Technol..

[137] Shukla P., Sakure A., Basaiawmoit B., Khakhariya R., Maurya R., Bishnoi M., Kondepudi K.K., Liu Z., Padhi S., Rai A.K. (2023). Molecular binding mechanism and novel antidiabetic and anti-hypertensive bioactive peptides from fermented camel milk with anti-inflammatory activity in raw macrophages cell lines. Amino Acids.

[138] Khakhariya R., Sakure A.A., Maurya R., Bishnoi M., Kondepudi K.K., Padhi S., Rai A.K., Liu Z., Patil G.B., Mankad M. (2023). A comparative study of fermented buffalo and camel milk with anti-inflammatory, ACE-inhibitory and anti-diabetic properties and release of bio active peptides with molecular interactions: In vitro, in silico and molecular study. Food Biosci..

[139] Patel D., Sakure A., Lodha D., Basaiawmoit B., Maurya R., Das S., Liu Z., Padhi S., Rai A.K., Hati S. (2021). Significance of Lactobacillus fermentum on antioxidative and anti-inflammatory activities and ultrafiltration peptide fractions as potential sources of antioxidative peptides from fermented camel milk (Indian breed). J. Am. Nutr. Assoc..

[140] Shukla P., Sakure A., Pipaliya R., Basaiawmoit B., Maurya R., Bishnoi M., Kondepudi K.K., Hati S. (2022). Exploring the potential of *Lacticaseibacillus paracasei* M11 on antidiabetic, anti-inflammatory, and ACE inhibitory effects of fermented dromedary camel milk (*Camelus dromedaries*) and the release of antidiabetic and anti-hypertensive peptides. J. Food Biochem..

[141] Dharmisthaben P., Sakure A., Liu Z., Maurya R., Das S., Basaiawmoit B., Kumari R., Bishnoi M., Kondepudi K.K., Gawai K.M. (2023). Identification and molecular mechanisms of novel antioxidative peptides from fermented camel milk (Kachchi breed, India) with anti-inflammatory activity in raw macrophages cell lines. Int. J. Dairy Technol..

[142] Ming L., Qi B., Hao S., Ji R. (2021). Camel milk ameliorates inflammatory mechanisms in an alcohol-induced liver injury mouse model. Sci. Rep..

[143] Zhu C., Sun W., Luo Y. (2024). Ameliorative effects of camel milk and fermented camel milk on acute alcoholic liver injury. Fermentation.

[144] Arab H.H., Salama S.A., Maghrabi I.A. (2018). Camel milk attenuates methotrexate-induced kidney injury via activation of PI3K/Akt/eNOS signaling and intervention with oxidative aberrations. Food Funct..

[145] Arab H.H., Salama S.A., Eid A.H., Omar H.A., Arafa E.-S.A., Maghrabi I.A. (2014). Camel’s milk ameliorates TNBS-induced colitis in rats via downregulation of inflammatory cytokines and oxidative stress. Food Chem. Toxicol..

[146] Mohamed W.A., Schaalan M.F. (2018). Antidiabetic efficacy of lactoferrin in type 2 diabetic pediatrics; controlling impact on PPAR-γ, SIRT-1, and TLR4 downstream signaling pathway. Diabetol. Metab. Syndr..

[147] Badr G., Abdel-Tawab H.S., Ramadan N.K., Ahmed S.F., Mahmoud M.H. (2018). Protective effects of camel whey protein against scrotal heat-mediated damage and infertility in the mouse testis through YAP/Nrf2 and PPAR-gamma signaling pathways. Mol. Reprod. Dev..

[148] Jing X., Du D., Hou B., Zhan D., Hasi S. (2024). Camel whey protein attenuates acute heat stress-induced kidney injury in rats by up-regulating CYP2J activity and activating PI3K/AKT/eNOS to inhibit oxidative stress. Vet. Sci..

[149] Behrouz S., Mohammadi M., Sarir H., Boskabady M.H. (2024). The effects of camel milk in systemic inflammation and oxidative stress of cigarette smoke-induced chronic obstructive pulmonary disease model in rat. Front. Vet. Sci..

[150] Kilari B.P., Mudgil P., Azimullah S., Bansal N., Ojha S., Maqsood S. (2021). Effect of camel milk protein hydrolysates against hyperglycemia, hyperlipidemia, and associated oxidative stress in streptozotocin (STZ)-induced diabetic rats. J. Dairy Sci..

[151] Althwab S.A., Alamro S.A., Al Abdulmonem W., Allemailem K.S., Alarifi S.A., Hamad E.M. (2022). Fermented camel milk enriched with plant sterols improves lipid profile and atherogenic index in rats fed high-fat and-cholesterol diets. Heliyon.

[152] Hassaneen N.H., Hemeda S.A., El Nahas A.F., Albadrani G.M., Al-Ghadi M.Q., Mohammedsaleh Z.M., Fadl S.E., El-Diasty E.M., Sakr H.I. (2025). Post-treatment of rat aflatoxicosis by camel milk and silymarin. Front. Pharmacol..

[153] Hassaneen N.H., Hemeda S.A., El Nahas A.F., Fadl S.E., El-Diasty E.M. (2023). Ameliorative effects of camel milk and silymarin upon aflatoxin B1 induced hepatic injury in rats. Sci. Rep..

[154] Hassaneen N.H., Hemeda S.A., El Nahas A.F., Fadl S.E., El-Diasty E.M. (2024). Camel milk or silymarin could improve the negative effects that experimentally produced by aflatoxin B1 on rat’s male reproductive system. BMC Vet. Res..

[155] Alshuniaber M.A., Alshammari G.M., Eleawa S.M., Yagoub A.E., Al-Khalifah A.S., Alhussain M.H., Al-Harbi L.N., Yahya M.A. (2022). Camel milk protein hydrosylate alleviates hepatic steatosis and hypertension in high fructose-fed rats. Pharm. Biol..

[156] Sunita Meena S.M., Rajput Y.S., Pandey A.K., Rajan Sharma R.S., Raghvendar Singh R.S. (2016). Camel milk ameliorates hyperglycaemia and oxidative damage in type-1 diabetic experimental rats. J. Camel Pract. Res..

[157] Du D., Lv W., Su R., Yu C., Jing X., Bai N., Hasi S. (2021). Hydrolyzed camel whey protein alleviated heat stress-induced hepatocyte damage by activated Nrf2/HO-1 signaling pathway and inhibited NF-κB/NLRP3 axis. Cell Stress. Chaperones.

[158] Du D., Lv W., Jing X., Ma X., Wuen J., Hasi S. (2021). Dietary supplementation of camel whey protein attenuates heat stress-induced liver injury by inhibiting NLRP3 inflammasome activation through the HMGB1/RAGE signalling pathway. J. Funct. Foods.

[159] Du D., Lv W., Jing X., Yu C., Wuen J., Hasi S. (2022). Camel whey protein alleviates heat stress-induced liver injury by activating the Nrf2/HO-1 signaling pathway and inhibiting HMGB1 release. Cell Stress Chaperones.

[160] Xu R.H., Xiu L., Zhang Y.L., Du R.P., Wang X. (2019). Probiotic and hepatoprotective activity of lactobacillus isolated from Mongolian camel milk products. Benef. Microbes.

[161] Seifu E. (2022). Recent advances on camel milk: Nutritional and health benefits and processing implications—A review. AIMS Agric. Food.

[162] Muthukumaran M.S., Mudgil P., Baba W.N., Ayoub M.A., Maqsood S. (2023). A comprehensive review on health benefits, nutritional composition and processed products of camel milk. Food Rev. Int..

[163] Al-Ghumlas A.K., Alhakbany M.A., Korish A.A. (2023). Antiapoptotic and anticoagulant effects of camel milk and camel urine in methotrexate-induced hepatotoxicity. CyTA J. Food.

[164] Mohamed H., Ranasinghe M., Amir N., Nagy P., Gariballa S., Adem A., Kamal-Eldin A. (2022). A study on variability of bioactive proteins in camel (*Camelus dromedarius*) milk: Insulin, insulin-like growth factors, lactoferrin, immunoglobulin G, peptidoglycan recognition protein-1, lysozyme and lactoperoxidase. Int. J. Dairy Technol..

[165] Ashraf A., Mudgil P., Palakkott A., Iratni R., Gan C.Y., Maqsood S., Ayoub M.A. (2021). Molecular basis of the anti-diabetic properties of camel milk through profiling of its bioactive peptides on dipeptidyl peptidase IV (DPP-IV) and insulin receptor activity. J. Dairy Sci..

[166] Izadi A., Khedmat L., Mojtahedi S.Y. (2019). Nutritional and therapeutic perspectives of camel milk and its protein hydrolysates: A review on versatile biofunctional properties. J. Funct. Foods.

[167] Solanki D., Hati S. (2018). Fermented camel milk: A review on its bio-functional properties. Emir. J. Food Agric..

[168] AlKurd R., Hanash N., Khalid N., Abdelrahim D.N., Khan M.A., Mahrous L., Radwan H., Naja F., Madkour M., Obaideen K. (2022). Effect of camel milk on glucose homeostasis in patients with diabetes: A systematic review and meta-analysis of randomized controlled trials. Nutrients.

[169] Abd-Elhakim Y.M., El-Sharkawy N.I., Mohammed H.H., Ebraheim L.L., Shalaby M.A. (2020). Camel milk rescues neurotoxic impairments induced by fenpropathrin via regulating oxidative stress, apoptotic, and inflammatory events in the brain of rats. Food Chem. Toxicol..

[170] Mohamed A.A.R., Abdellatief S.A., Khater S.I., Ali H., Al-Gabri N.A. (2019). Fenpropathrin induces testicular damage, apoptosis, and genomic DNA damage in adult rats: Protective role of camel milk. Ecotoxicol. Environ. Saf..

[171] Shaban A.M., Raslan M., Sharawi Z.W., Abdelhameed M.S., Hammouda O., El-Masry H.M., Elsayed K.N., El-Magd M.A. (2023). Antibacterial, antifungal, and anticancer effects of camel milk exosomes: An in vitro study. Vet. Sci..

[172] Korish A.A., Arafah M.M. (2013). Camel milk ameliorates steatohepatitis, insulin resistance and lipid peroxidation in experimental non-alcoholic fatty liver disease. BMC Complement. Altern. Med..

[173] Ebaid H., Abdel-Salam B., Hassan I., Al-Tamimi J., Metwalli A., Alhazza I. (2015). Camel milk peptide improves wound healing in diabetic rats by orchestrating the redox status and immune response. Lipids Health Dis..

